# Clinical outcomes associated with anti‐obesity medications in real‐world practice: A systematic literature review

**DOI:** 10.1111/obr.13326

**Published:** 2021-08-22

**Authors:** Nadia N. Ahmad, Susan Robinson, Tessa Kennedy‐Martin, Jiat Ling Poon, Hong Kan

**Affiliations:** ^1^ Research and Development – Obesity, Lilly Diabetes Eli Lilly and Company Indianapolis IN USA; ^2^ Kennedy‐Martin Health Outcomes Ltd Hove UK; ^3^ Global Patient Outcomes and Real World Evidence Eli Lilly and Company Indianapolis IN USA

**Keywords:** anti‐obesity medication, effectiveness

## Abstract

Anti‐obesity medications (AOMs) are efficacious and well tolerated in randomized controlled trials, but findings may not be generalizable to routine clinical practice. This systematic literature review aimed to identify real‐world (RW) evidence for AOMs to treat adults ( ≥ 18 years) with obesity or overweight (BMI  ≥  27 kg/m^2^). Searches conducted in MEDLINE, Embase, Health Technology Assessment (HTA) Database, National Health Service (NHS) Economic Evaluation Database, and Cochrane Central Register of Controlled Trials for studies of relevant FDA‐approved AOMs yielded 41 publications. Weight loss (WL) was consistently observed, with 14% to 58.6% of patients achieving ≥ 5% WL on orlistat, phentermine/topiramate, naltrexone/bupropion, phentermine, or liraglutide in studies of 3–6 months' duration where this was measured. When cardiometabolic risk factors were assessed, AOMs reduced or had no impact on blood pressure, lipids, or glycemia. RW data on the impact of AOMs on existing obesity‐related comorbidities and mortality were generally lacking. AOMs were associated with various adverse events, but these were of mild to moderate severity and no unexpected safety signals were reported. A pattern of poor adherence and persistence with AOMs was observed across studies. Overall, the review confirmed the effectiveness of AOMs in RW settings but demonstrated large gaps in the evidence base.

**Abbreviations**
AE: adverse event
AERS: adverse event reporting system
ALT: alanine transaminase
AOM: anti‐obesity medication
BMI: body mass index
BPN: bupropion
CEA: cost‐effectiveness analysis
CI: confidence interval
CPRD: Clinical Practice Research Database
CVD: cardiovascular disease
DBP: diastolic blood pressure
DM: diabetes mellitus
EMR: electronic medical records
FBG: fasting blood glucose
FDA: Food and Drug Administration
FEN: fenfluramine
HbA_1c_
: glycated hemoglobin
HCRU: healthcare resource utilization
HDL‐C: high‐density lipoprotein cholesterol
HMO: Health Management Organization; HR, heart rate
HRQoL: health‐related quality of life
hsCRP: high‐sensitivity C‐reactive protein
HTA: Health Technology Assessment
IQR: interquartile range
LAGB: laparoscopic‐adjustable gastric band
LDL‐C: low‐density lipoprotein cholesterol
LIRA: liraglutide
LORC: lorcaserin
MACE: major adverse cardiovascular event
MI: myocardial infarction
MPR: medication possession ratio
NHS: National Health Service
NTX: naltrexone
OECD: Organisation for Economic Co‐operation and Development
OR: odds ratio
ORL: orlistat
PCMH: patient‐centered medical home
PDC: proportion of days covered
PHEN: phentermine
PORTAL: Patient Outcomes Research to Advance Learning
PRO: patient‐reported outcome
PSM: propensity score matched
RCT: randomized controlled trial
RW: real‐world
RWE: real‐world evidence
RYGB: Roux‐en‐Y gastric bypass
SAE: serious adverse event
SBP: systolic blood pressure
SG: sleeve gastrectomy
SIB: sibutramine
T2DM: type 2 diabetes mellitus
TC: total cholesterol
TG: triglycerides
TPM: topiramate
VA: Veteran's Affairs
VLED: very‐low energy diet
WL: weight loss

## 1 INTRODUCTION

Obesity is a major public health issue with a prevalence that has tripled over the last 45 years.^1^ In 2015, it was estimated that nearly 604 million adults (12%) worldwide were classified as having obesity (body mass index [BMI]  ≥  30 kg/m^2^).^2^ Furthermore, in an analysis of the 2015 Global Burden of Disease study, high BMI was reported to account for 4 million deaths globally and to contribute to 120 million disability‐adjusted life years.^2^ Obesity also imposes a considerable economic burden on healthcare systems and society,^3^ primarily driven by the treatment of obesity‐related chronic diseases as well as presenteeism, absenteeism, and reduced employment rates.^4^ For example, in the United States, individuals with obesity had annual healthcare costs US$3500 higher than individuals without obesity, resulting in a national cost of US$316 billion per year or 27.5% of US healthcare spending in 2010.^5^ Similarly, international data from 52 Organisation for Economic Co‐operation and Development (OECD) countries suggests that over the next 30 years, overweight and obesity will cost US$425 billion per year, representing 8.4% of total global healthcare spending.^4^

Prevention of obesity through policy changes and healthy lifestyle promotion is critical to curb the worsening epidemic. However, with such high proportions of individuals already manifesting obesity, there is also a pressing need for treatment. A stepwise approach to obesity treatment is generally advocated involving initial lifestyle interventions followed by pharmacologic intervention and bariatric surgery, if necessary. Lifestyle‐based therapies represent the cornerstone of obesity management, but alone do not provide sustainable weight loss in most individuals,^6^ and bariatric surgery, though highly effective, is applied in only a minority of eligible cases.^7^ As such, there is an urgent need for well‐tolerated and effective pharmacologic anti‐obesity therapy. Currently, five anti‐obesity medications (AOMs; liraglutide 3 mg, semaglutide 2.4 mg, orlistat, naltrexone/bupropion [NTX/BPN], and phentermine/topiramate [PHEN/TPM]) are approved for long‐term use by the US Food and Drug Administration (FDA) for the treatment of adults with a BMI  ≥  30 or ≥  27 kg/m^2^ with at least one weight‐related comorbidity, and several other medications are in clinical development.^8^, ^9^ Furthermore, another four treatments (phentermine, benzphetamine, diethylpropion, and phendimetrazine) are FDA approved for short‐term (a few weeks) use, although with the exception of phentermine these are rarely utilized in real‐world settings.^10^

The efficacy and safety of AOMs have been well documented in randomized controlled trials (RCTs). A systematic literature review including 35 RCTs reported that the AOMs FDA‐approved for long‐term use at the time were all associated with greater weight loss and weight‐loss maintenance compared with placebo and were associated with generally low rates of serious adverse events (SAEs).^11^ However, the effectiveness of AOMs in real‐world practice is not as well understood. Unlike RCTs, real‐world studies include heterogeneous patient samples that are more representative of the general disease population likely to be treated by primary care and specialist physicians. Real‐world studies can support data from RCTs and provide more information on clinical outcomes, safety signals, patient persistence and adherence, economic outcomes, and longer‐term treatment trends, all of which are fundamental in informing disease management practices and healthcare policy.^12^

The objective of the current review was, therefore, to identify, summarize, and interpret retrospective or prospective published studies that provide real‐world evidence (RWE) for AOMs in the treatment of adults ( ≥ 18 years) with obesity or overweight. While the original search comprised a broad focus, this manuscript is limited to a summary of weight change, cardiometabolic risk factors, adverse events (AEs), and adherence, persistence, and discontinuation, since these were the most commonly and consistently reported measures.

## 2 METHODS

A robust and reproducible protocol for the literature search was developed that detailed the proposed approach, objectives, search strategy, study selection criteria, methods for data extraction and synthesis, and outcomes of interest that were specified a priori. The protocol reduced the potential impact of review author bias, ensured transparency and accountability, and maximized the chances of accurate data extraction.

### 2.1 Search strategy

MEDLINE, Embase, the Health Technology Assessment (HTA) Database, the National Health Service (NHS) Economic Evaluation Database, and the Cochrane Central Register of Controlled Trials were searched to identify relevant studies. Searches were run in October 2019 with no date limit. A hand search of the bibliographies of eligible publications was also undertaken to identify any relevant studies that were not found by the original search.

The overall search strategy comprised three concepts: “weight loss” AND “specific AOMs of interest” AND “RWE.” Notably, the more general concept of “AOMs” without mention of specific drugs of interest was not a part of the search strategy as the aim was to only identify and include studies in which drug‐level data for the specific AOMs of interest were presented. Concepts were captured using subject headings and text‐word searches in the title, abstract, and keyword‐heading fields. A base‐case strategy was developed for MEDLINE and adapted to the other databases (Tables S1–S5); additional details regarding the search strategy can be found in the Supporting Information.

### 2.2 Eligibility criteria

The search eligibility criteria are shown in Table 1. While the original search included a range of AOMs, only those that were FDA‐approved for long‐term use at the time of the search are the focus of the current article. Publications that evaluated outcomes associated with lorcaserin and sibutramine are not summarized here, but where evaluated as comparators in the included studies, findings were noted. Of the AOMs FDA‐approved for short‐term use, only phentermine was included as it is one of the most frequently prescribed in real‐word practice.^10^

**TABLE 1:** Study selection criteria

Study characteristic	Eligibility criteria
Patient population	▪ Adults (age ≥ 18 years) with overweight or obesity
AOM	▪ Orlistat ▪ Lorcaserin hydrochloride^a^ ▪ Phentermine ▪ Phentermine–topiramate ▪ Naltrexone–bupropion ▪ Liraglutide ▪ Sibutramine^a^
Comparator	▪ All interventions including placebo or usual care and other AOMs ▪ Baseline (before/after comparison) ▪ No comparator
Outcomes^b^	▪ Weight change (BMI, total fat mass, visceral fat mass, weight, waist circumference, waist:hip ratio) ▪ Cardiometabolic risk factors (lipids, hsCRP, ALT, SBP, DBP, HbA_1c_, FBG, fasting insulin) ▪ Incidence of obesity‐related comorbidities ▪ Change in existing comorbidity ▪ Adverse events ▪ Economic outcomes (costs, healthcare resource utilization) ▪ Patient‐reported outcomes (HRQoL, patient preference, patient satisfaction, functioning/activities of daily living, pain and discomfort) ▪ Adherence, persistence, discontinuation, reasons for discontinuation
Study type	▪ RW cross‐sectional ▪ RW case–control ▪ RW cohort ▪ Pragmatic clinical trials ▪ Administrative or claims database ▪ Electronic medical records ▪ Registry representing RW practice ▪ Questionnaires and surveys relating to RW practice
Language	▪ English

Abbreviations: ALT, alanine transaminase; AOM, anti‐obesity medication; BMI, body mass index; DBP, diastolic blood pressure; FBG, fasting blood glucose; HbA_1c_, glycated hemoglobin; HCRU, healthcare resource utilization; HRQoL, health‐related quality of life; hsCRP, high‐sensitivity C‐reactive protein; RW, real‐world; SBP, systolic blood pressure.

^a^ Sibutramine and lorcaserin are not FDA‐approved but were included in the broad search; studies including only data related to these drugs were excluded from the review.

^b^ Only most commonly and consistently reported outcomes described in the review; for example, economic outcomes and patient‐reported outcomes were rarely included and so findings are not reported in this review.

### 2.3 Study selection process

Search results were assessed independently by two reviewers, using a two‐phase approach that consisted of (1) a broad review of the title and/or abstract of search results and (2) a subsequent full‐text review of potentially eligible studies identified at Stage 1. Any studies failing to meet the selection criteria at Stage 2 were excluded and the reason for exclusion recorded. Any disagreements between reviewers were resolved by discussion until consensus was met.

Data extraction was performed on a standardized data extraction form by two reviewers, with quality checking by a third. Variables extracted included study population, interventions, study type and methods (including data source), study duration, and specific outcomes data.

## 3 RESULTS

**FIGURE 1:**
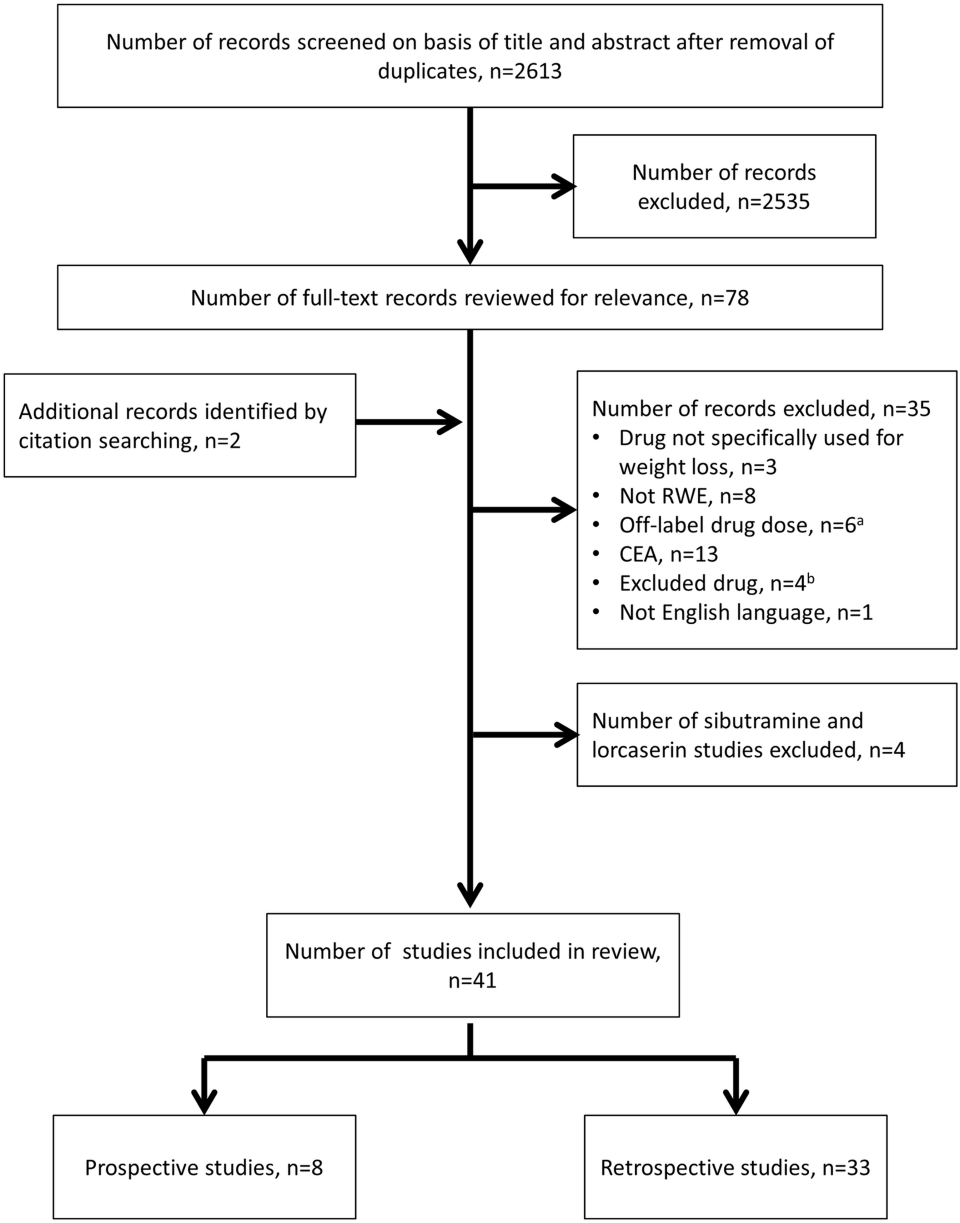
Study selection. ^a^Studies including liraglutide not used at 3.0 mg dose for weight loss. ^b^Studies included the following non‐specified AOMs: PHEN/FEN, PHEN/FLU, mazindol, and caffeine/ephedrine, and a study that pooled 15 AOMs of which only five were eligible for this review. AOM, anti‐obesity medication; CEA, cost‐effectiveness analysis; FEN, fenfluramine; FLU, flunarizine; PHEN, phentermine; RWE, real‐world evidence

The search identified 2613 studies for eligibility review after removal of duplicates, of which 2535 were excluded following review of titles and abstracts. Of 78 full‐text records, 35 were excluded (Figure 1). An additional two studies were identified by citation searching of included records to yield a total of 45 studies. Four of these studies evaluated sibutramine alone and so were also excluded, leaving 41 eligible studies for inclusion in the review.

### 3.1 Study characteristics

Table 2 provides an overview of the characteristics of included studies. Studies were conducted across a wide geography, with the United States and the United Kingdom being the most represented countries.

**TABLE 2:** Characteristics of included studies, by study design and alphabetical by author

Author (country)	Study type/patient population	Healthcare setting^a^	Data source	Baseline characteristics of study population^b^			Study drug(s), *n*	Comparator	Background measures described, Y/N^c^	Outcome(s) studied						
				Age, years (mean or median)	Male, %	BMI, kg/m^2^ weight, kg				Weight	Cardiometabolic risk factors	Existing comorbidity	AEs	Economic	PRO	Adherence
**PROSPECTIVE STUDIES**																
Hayes et al. (UK)^13^	Prospective	P	EMR (CPRD)	46.1	ORL: 24.1 SIB: 17.6	BMI: 36.4	ORL, *n* = 77,047	SIB, *n* = 23,927	N				✓			
Hollywood and Ogden (UK)^14^	Prospective	P	Questionnaire (Xenical Support System)	50.2	17.5	BMI: 36.2	ORL, *n* = 566	Baseline (pre‐drug)	Y	✓						✓
Kim et al. (Korea)^15^	Prospective	P	Clinic visits (multicenter)	35.6	5.4	BMI: M, 31.5; F, 26.8; Weight: M, 94.2; F, 67.8	PHEN, *n* = 795	Baseline (pre‐drug)	N	✓	✓		✓			✓
Rowe et al. (UK)^16^	Prospective, DM	S	Clinic visit (single center)	M, 54.5 F, 54.8	45	BMI: 39.5 Weight: M, 126.2; F, 98.1	ORL, *n* = 100	Baseline (pre‐drug)	Y	✓	✓	✓		✓		✓
Schwartz et al. (USA)^17^	Prospective	P, S	Pharmacy visits/telephone interview	44.9	14.3	BMI:32.0 Weight: 88.6	ORL, *n* = 237	Baseline (pre‐drug)	Y	✓			✓		✓	✓
Wirth (Germany)^18^	Prospective	P	Clinic visits (multicenter)	48	28.4	BMI: 34.7 Weight: 99.2	ORL, *n* = 15,549 (alone + adjunctive measures)	Baseline (pre‐drug)	Y	✓	✓	✓	✓		✓	✓
**PROSPECTIVE STUDIES INCLUDING POSTSURGICAL PATIENTS**																
Suliman et al. (UAE)^19^	Prospective, surgical and non‐surgical	S	Clinic visits (single center)	39	20	BMI: 37.5 Weight: 97.9	LIRA, *n* = 787 (surgical, *n* = 76; non‐surgical, *n* = 711)	Baseline (pre‐drug)	N	✓			✓			✓
Wharton et al. (Canada)^20^	Prospective, surgical	S	Clinic visits (single center)	51.2	12.8	BMI: 42.5	LIRA, *n* = 117	Baseline (pre‐drug)	Y	✓			✓			✓
**RETROSPECTIVE STUDIES**																
Aagaard et al. (Europe)^21^	Retrospective	P, S	AERS	> 18 y, 98%	25^b^		ORL, *n* = 1710^d^ PHEN, *n* = 15 SIB, *n* = 626	None	N				✓			
Acharya et al. (UK)^22^	Retrospective	P	Prescription data	45	19.9		ORL, *n* = 16,021	None	N				✓			✓
Ahn et al. (Korea)^23^	Retrospective	S	Medical records (single center)	47.5	15.9	BMI: 30.6 Weight: 79.3	ORL, *n* = 63	Baseline (pre‐drug)	Y	✓	✓		✓			
Allie et al. (USA)^24^	Retrospective, T2DM	S	EMR (single center)	53	41.5	BMI: 40 Weight: 116	ORL, *n* = 41	Baseline (pre‐drug)	N	✓	✓	✓				✓
Beermann et al. (Sweden)^25^	Retrospective	P, S	Postal questionnaire	50	29.9	BMI: 35.0 Weight: 99.3	ORL after ≥ 2.5 kg, *n* = 156 or < 2.5 kg diet weight loss, *n* = 107	Baseline (pre‐drug)	Y	✓						✓
Costello et al. (USA)^26^	Retrospective	P	EMR (PCMH model)	34	7.4	BMI: 40.7 Weight: 113.6	PHEN, *n* = 22 PHEN/TPM, *n* = 5	Baseline (pre‐drug)	Y	✓						
Czernichow et al. (The Netherlands)^27^	Retrospective	P, S	Registry (pharmacy data)	46.5	21.3		ORL, *n* = 6139	Baseline (3 years pre‐drug)	N			✓				
Derby et al. (UK)^28^	Retrospective	P	EMR (CPRD)				PHEN, *n* = 887	Matched obese, no AOM, *n* = 17,225	N				✓			
Douglas et al. (UK)^29^	Retrospective	P	EMR (CPRD)	48.4	31.3		ORL, *n* = 988	Non‐use period (within patient comparison)	N				✓			
Douglas et al. (UK)^30^	Retrospective	P	EMR (CPRD)	46.2	23.6	BMI: 37.2	ORL, *n* = 100,701 SIB, *n* = 15,355	Baseline (pre‐drug) Matched obese non‐exposed, *n* = 508,140	Y	✓						
Ganguly et al. (USA)^31^	Retrospective	P, S	Claims data (Truven Health)	46.7–48.5	17.8–27.6		LIRA, *n* = 4083 NTX/BPN, *n* = 11,660 PHEN/TPM, *n* = 4195 LORC, *n* = 6584	Between drugs	N							✓
Gorgojo‐Martínez et al. (Spain)^32^	Retrospective	S	EMR (single center)	ORL, 47.3 LIRA, 51.9	ORL, 25 LIRA, 27	ORL, BMI: 41.4; weight: 107.8 LIRA, BMI: 39.7; weight: 105.1	ORL, *n* = 400 LIRA, *n* = 100	Baseline (pre‐drug) Between drugs	Y	✓	✓	✓	✓			✓
Grabarczyk (USA)^33^	Retrospective	P, S	EMR (VA Corporate Data Warehouse)	52.4–58.4	62.5–76.5	BMI: 39.5–41.5 Weight: 121.1–128.0	ORL, *n* = 6153 LORC, *n* = 298 PHEN, *n* = 304 PHEN/TPM, *n* = 233	Baseline (pre‐drug) Between drugs Weight‐management program, *n* = 59,047	Y	✓	✓					✓
Graham et al. (USA)^34^	Retrospective, T2DM	P, S	EMR (single center)	ORL, 54.6	93	BMI: 48.3 Weight: 152.0	ORL, *n* = 29 ORL + WL clinic, *n* = 18	Baseline (pre‐drug)	Y	✓	✓		✓			✓
Hemo et al. (Israel)^35^	Retrospective	P, S	EMR (HMO)	21–40 y, 42.4% 41–60 y, 49.5% > 60 y, 8.1%	20.9	BMI: 34.4	ORL + SIB, *n* = 1340/*n* = 5501 (Data are pooled)	Baseline (pre‐drug)	N	✓						✓
Hendricks et al. (USA)^36^	Retrospective	S	Medical records (single center)	49.6	15	BMI: 35.6 Weight: 98.8	PHEN + WL program, *n* = 269 WL program only, *n* = 31	Baseline (pre‐drug or pre‐program)	Y	✓	✓		✓			
Hong et al. (UK)^37^	Retrospective	P	EMR (CPRD)	47	22.6	BMI: 36	ORL, *n* = 33,625	Matched obese non‐initiators, *n* = 160,347	N				✓			
Horie et al. (Brazil)^38^	Retrospective, ≥ 60 years	S	Medical records (single center)	65.2	14	BMI: 38.5 Weight: 95.3	ORL, *n* = 11 SIB, *n* = 29	Baseline (pre‐drug)	N	✓						✓
Jick et al. (UK)^39^	Retrospective	P	EMR (CPRD)	NR			PHEN, *n* = 862	Matched obese non‐initiators, *n* = 9281	N				✓			
Lewis et al. (USA)^40^	Retrospective	P, S	EMR (PORTAL cohort)	43.5	16.3	BMI: 37.8	PHEN, *n* = 13,972	Baseline (pre‐drug) Time‐varying PHEN exposure categories	N	✓	✓		✓			
Li et al. (USA)^41^	Retrospective	S	Medical records (single center)	M: 45.5 F: 46.7	31.9	BMI: 38.7 (M), 38.1 (F) Weight: 124.9 (M); 103.4 (F)	PHEN, *n* = 188	Baseline (pre‐drug)	Y	✓			✓			✓
Neoh et al. (Australia)^42^	Retrospective	S	EMR (single center)	49	36.9	BMI: 48.6 Weight: 135.5	PHEN/TPM, *n* = 103	Pre‐VLED/pre‐drug End VLED/pre‐drug	Y	✓	✓		✓			
Padwal et al. (Canada)^43^	Retrospective	P, S	Claims data (PharmaNet and British Colombia Linked Health)	45	20		ORL, *n* = 16,968 SIB, *n* = 3466	None	N							✓
Perrio et al. (UK)^44^	Retrospective	P	Prescription records	45	19.4		ORL, *n* = 16,021 SIB, *n* = 12,336	None	N				✓			✓
Poongodi and Parthiban (India)^45^	Retrospective	P, S	AERS	NR			ORL	None	N				✓			
Ritchey et al. (USA)^46^	Retrospective	P, S	Claims data (Truven Health)	43.8–47.3	17.2–20.3		PHEN/TPM, *n* = 14,586 PHEN/TPM (individual use), *n* = 19,184 PHEN, *n* = 124,334	Periods of non‐drug use in former users	N				✓			
Shibuya et al. (USA)^47^	Retrospective	P, S	EMR (Cleveland Clinic)	44.4–51.9	14.7–18.1	BMI: 37.7–39.4 Weight: 104.1–110.5	PHEN/TPM, *n* = 425 PHEN, *n* = 2372 NTX/BPN, *n* = 260 LORC, *n* = 354	Baseline (pre‐drug) Between drugs	Y	✓						
Wharton et al. (Canada)^48^	Retrospective	S	EMR (multicenter)	49.7	17	BMI: 40.7 Weight: 114.8	LIRA, *n* = 311	Baseline (pre‐drug)	Y	✓	✓					✓
**RETROSPECTIVE STUDIES INCLUDING POSTSURGICAL PATIENTS**																
Elhag et al. (Qatar)^49^	Retrospective Surgical and non‐surgical	S	EMR (single center)	Non‐surgical, 40.9 Surgical, 45.1	Non‐surgical, 22.9 Surgical, 5.3	Non‐surgical, BMI: 38.3; Weight: 100.2 Surgical, BMI: 37.0; Weight: 94.8	PHEN non‐surgical, *n* = 48; surgical, *n* = 19	Baseline (pre‐drug) LORC (surgical and non‐surgical)	Y	✓	✓		✓	✓		
Nor Hanipah et al. (USA)^50^	Retrospective, surgical	S	Medical records (single center)	50.9	6.7	BMI: 40.2 (LABG); 37.3 (RYGB); 36.9 (SG) Weight, 106.5^e^ (LABG); 100.5 (RYGB); 101.2 (SG)	PHEN/TPM, *n* = 25 PHEN, *n* = 156 LORC, *n* = 18 NTX/BPN, *n* = 10 (data are pooled)	Baseline (pre‐drug)	Y	✓						
Rye et al. (Canada)^51^	Retrospective, surgical	S	Medical records (single center)	49.6	5	BMI ≥ 30 SG, 35% RYGB, 35% Other, 30%	LIRA, *n* = 20	Baseline (post‐surgery, pre‐drug)	Y	✓			✓			
Schwartz et al. (USA)^52^	Retrospective, surgical	S	Medical records (single center)	PHEN, 46.8 PHEN/TPM, 48.9	PHEN, 9.6 PHEN/TPM, 15.4	BMI: PHEN, 39.7 (PHEN); 37.9 (PHEN/TPM) Weight: 111.1^d^ (PHEN); 105.8 (PHEN/TPM)	PHEN, *n* = 52 PHEN/TPM, *n* = 13	Baseline (pre‐drug) Between drugs	Y	✓		✓				✓
Toth et al. (USA)^53^	Retrospective, surgical (21–30 years)	S	Medical records (multicenter)	21–25 y, 40.5% ≥ 26 y, 59.5%	8.1	BMI: 38.5^d^	PHEN, *n* = 37^f^	Baseline (pre‐drug)	N	✓						

Abbreviations: AE, adverse event; AERS, adverse event reporting system; AOM, anti‐obesity medication; BMI, body mass index; BPN, bupropion; CPRD, Clinical Practice Research Database; DM, diabetes mellitus; EMR, electronic medical records; HMO, Health Management Organization; LAGB, laparoscopic‐adjustable gastric band; LIRA, liraglutide; LORC, lorcaserin; M, male; NR, not reported; NTX, naltrexone; ORL, orlistat; P, primary care; PCMH, patient‐centered medical home; PHEN, phentermine; PORTAL, Patient Outcomes Research to Advance Learning; PRO, patient‐reported outcome; RYGB, Roux‐en‐Y gastric bypass; S, secondary care; SG, sleeve gastrectomy; SIB, sibutramine; T2DM, type 2 diabetes mellitus; TPM, topiramate; VA, Veteran's Affairs; VLED, very‐low energy diet; WL, weight loss.

^a^ Secondary care also includes tertiary hospitals.

^b^ Where a range of values are provided, this reflects multiple treatment groups.

^c^ Where described, type of background measure varied across studies but included participation in weight‐loss programs, education and counseling on lifestyle measures, dietary changes including very‐low calorie diets, and increases in physical activity.

^d^ *n* = number of SAE reports, not number of patients.

^e^ Values are post‐surgery, but pre‐drug.

^f^ Sample size for all patients who received AOMs after surgery; number of patients receiving phentermine specifically was not reported.

#### 3.1.1 Study designs

Most studies were of a retrospective design (*n* = 33), and data were mostly collected from medical records and charts (electronic or otherwise). Other data sources utilized in retrospective studies included administrative claims databases, pharmacy prescription data, and AE reporting systems. Only eight studies were prospective in design, with data mostly obtained at prespecified clinic visits. Studies were conducted specifically in primary (*n* = 12) and secondary/tertiary care settings such as specialist clinics, academic centers, and hospitals (*n* = 16), with 13 studies including data from both settings. In most studies, outcomes associated with AOMs were compared with baseline (pre‐drug) measures. Few studies included direct comparisons between different AOMs and even fewer a direct comparison of AOMs with diet and lifestyle modifications.

#### 3.1.2 Adjunctive measures

As AOMs are recommended as an adjunct to diet and exercise, lifestyle‐based therapies were described in approximately half of studies, although the level of detail varied widely. Measures generally included counseling and education with respect to diet and lifestyle changes and/or participation in weight‐loss clinics or programs. In some studies, diet and lifestyle interventions preceded AOM use, while in others lifestyle intervention was delivered together with AOMs. Compliance with such measures was seldom captured.

#### 3.1.3 Study populations

A general population of individuals with obesity or overweight was evaluated in most studies. Three studies included patients with obesity and diabetes mellitus (predominantly type 2 diabetes mellitus [T2DM]).^16^, ^24^, ^34^ Weight outcomes were also evaluated in subgroup analyses in individuals with T2DM or cardiovascular disease (CVD) in two further studies.^30^, ^47^ A single study evaluated elderly patients with obesity.^38^

Several studies were also identified that included surgical patients who had undergone a variety of bariatric procedures including Roux‐en‐Y gastric bypass (RYGB), sleeve gastrectomy (SG), laparoscopic adjustable gastric band (LAGB), and vertical banded gastroplasty.^19^, ^20^, ^49^, ^50^, ^51^, ^52^ Patients in these studies received AOMs if they had regained weight from their postsurgical nadir weight or experienced inadequate initial weight loss following bariatric surgery. One study specifically included a younger (21–30 years) postsurgical population,^53^ and one included a subgroup analysis in patients with T2DM.^51^

#### 3.1.4 Study drugs and outcomes evaluated

Orlistat was the most evaluated AOM across studies (*n* = 21), followed by phentermine (*n* = 14), PHEN/TPM (*n* = 7), liraglutide (*n* = 6), and NTX/BPN (*n* = 2). Weight change was the most studied outcome (*n* = 28), followed by AEs (*n* = 24), adherence/persistence/discontinuation (*n* = 21), and cardiometabolic risk factors (*n* = 13).

### 3.2 Weight outcomes

Across the 28 studies including weight outcomes, regardless of study population, the most consistently reported measures were absolute weight reduction (in kg or lb; *n* = 22 studies), percentage reduction in body weight (*n* = 19), and categorical weight loss according to clinically meaningful thresholds (*n* = 15). The main findings from studies reporting on these measures are summarized in Table 3. Other weight outcomes included changes in BMI and waist‐to‐hip ratio, but these were seldom reported. Weight outcomes associated with orlistat (12 studies) or phentermine (10 studies) were the most reported; five studies reported on weight outcomes with PHEN/TPM and five with liraglutide, while only a single study included NTX/BPN (Table 3). Findings were pooled from multiple AOMs in two additional studies.^35^, ^50^

**TABLE 3:** Weight outcomes (body weight change and categorical body weight loss) reported in included studies

Study	Patient population	Baseline weight, kg	Weight change^a^		Categorical body weight loss
			kg	%	
**ORLISTAT: PROSPECTIVE COHORTS**					
Hollywood and Ogden^14^	General obesity	NR	−4.09 (6.21) kg at 6 months		
Schwartz et al.^17^		88.6 (19.5) kg	−4.6 (0.7) kg at > 60 days		> 5%: > 60 day, 46.3%
Wirth^18^		99.2 kg	−10.8 (6.9) kg at 6–9 months (all patients)	6–9 months: all patients, −10.7%; ORL alone, −9.4%; + WL program, −10.8%; +diet, −9.5%; + exercise, −10.6%; + diet + exercise, −11.4%; +all adjunct measures, −12.0%	5–10%: 6–9 months, 35.8% (all patients)
Rowe et al.^16^	DM (91% T2DM)	F, 98.1 (18.0) kg; M, 126.2 (23.7) kg	−7.1 kg at 6 months (*p* < 0.001 vs. baseline)	−6.2% at 6 months	> 5%: 6 months, 51.2%
**ORLISTAT: RETROSPECTIVE COHORTS**					
Ahn et al.^23^	General obesity	79.3 (14.1) kg	−3.0 (2.0) kg at 12 weeks; −3.6 (2.9) kg at 24 weeks (*p* < 0.001 vs. baseline both time points)	−3.8% at 12 weeks; −4.6% at 24 weeks (*p* < 0.001 vs. baseline both time points)	5–10%: 12 weeks, 22.2%; 24 weeks, 34.9%
Beerman et al.^25^		99.3 (17.7) kg	3 months: pre‐drug diet weight loss < 2.5 kg, −4.8 (3.4) kg; pre‐drug diet weight loss ≥ 2.5 kg, −5.4 (4.1) (*p* = NS between groups)	3 months: pre‐drug diet weight loss < 2.5 kg, −5.0%; pre‐drug diet weight loss ≥ 2.5 kg, −5.3%	5 to < 10%: 38.4% (all patients), 40.2% (pre‐drug diet loss < 2.5 kg), 36.5% (pre‐drug diet loss ≥ 2.5 kg) at 3 months
Douglas et al.^30^		NR	1–4 months, −0.94 kg/month; 5–25 months, +0.16 kg/month; 26–36 months, +0.01 kg/month Small progressive weight increases in matched, non‐exposed controls over 3 years		
Gorgojo‐Martínez et al.^32^		107.8 (19.1) kg	−3.8 kg at 3–6 months; −3.3 kg at end of follow‐up (*p* < 0.0001 vs. baseline both time points)		> 5%: 3–6 months, 30.7%; end of follow‐up, 27.4%
Grabarczyk^33^		121.2 (26.2) kg		−2.2 (12.8)% at 12 weeks; −2.1 (12.7)% at ≥ 20 weeks; −2.8 (14.5)% at 36 weeks	≥ 5%: 12 weeks, 23.5%; ≥ 20 weeks, 27.1%
Allie et al.^24^	T2DM	116 (24.6) kg	−6.0 kg at 3–6 months (*p* < 0.001 vs. baseline)	−5% at 3–6 months	
Douglas et al.^30^		NR	1–4 months, −0.78 kg/month; 5–25 months, +0.10 kg/month; 26–36 months, −0.03 kg/month		
Graham et al.^34^		ORL + WL clinic, 134.5 (23.6) kg; ORL alone, 152 (28) kg	6 months: ORL + WL clinic, −5.0 (6.0) kg (*p* = 0.005 vs. baseline); ORL alone, −2.2 (4.7) kg (*p* = 0.027 vs. baseline) (*p* = NS between groups)		≥ 5%: 28% (ORL + WL clinic), 14% (ORL alone) at 6 months (*p* = NS between groups)
Douglas et al.^30^	CVD^b^	NR	1–4 months, −0.97 kg/month; 5–25 months, +0.13 kg/month; 26–36 months, 0.00 kg/month		
Horie et al.^38^	≥ 60 years	96.2 (15.78) kg	−3.30 (5.86) kg at 9 months		
**PHENTERMINE: PROSPECTIVE COHORTS**					
Kim et al.^15^	General obesity	Men, 94.2 (18.3) kg; Women, 67.8 (11.6) kg	−3.8 (4.0) kg at 12 weeks	−5.2% at 12 weeks	≥ 5%: 12 weeks, 45.6%
**PHENTERMINE: RETROSPECTIVE COHORTS**					
Costello et al.^26^	General obesity	Median (range), 109.5 (75.9–156.6) kg	Median (range), −4.2 (−13.9 to 5.5) kg at 12 weeks	Median −4.9% at 12 weeks	≥ 5%: 12 weeks, 50%
Grabarczyk^33^		121.5 (29) kg		−2.1 (14.2)% at 12 weeks; −3.6 (11.9)% at ≥ 20 weeks; −2.5 (14.4)% at 36 weeks	≥ 5%: 12 weeks, 30.5%; ≥ 20 weeks, 38.5%
Hendricks et al.^36^		PHEN + WL program, 98.4 (24.5) kg; WL program, 102.7 (19.8) kg		12 weeks: PHEN + WL program, −15.1 (4.0)% vs. WL program, −12.8 (3.9)% 26 weeks: −18.9 (6.5)% vs. − 17.4 (6.0)% 52 weeks: −17.6 (7.8)% vs. − 16.1 (9.7)% 104 weeks: −12.7 (8.2)% vs. − 8.4 (10.7)% (*p* = 0.0144 between groups)	≥ 5%: 97% (PHEN), 80% (WL program only) at 52 weeks
Lewis et al.^40^		NR		6 months: short‐term use, −2.7%; medium‐term use,−7.7%^c^ 12 months: −1.4%; −6.0% 24 months: −0.2%; −1.9%; long‐term use, −7.5%^c^	
Li et al.^41^		Men, 124.9 (SEM, 28.2) kg; Women, 103.4 (24.0) kg	−7.6 (4.4) kg (men), −6.3 (3.4) kg (women) at 8 weeks −9.7 (5.3) kg (men), −8.0 (6.5) kg (women) at 12 weeks		
Shibuya et al.^47^	104.7 (24.8) kg	−3.87 (5.9) kg at 12 weeks (*p* < 0.0001 vs. baseline)	−3.75% at 12 weeks	≥ 5%: 12 weeks, 39%
Elhag et al.^49^	Surgical	Surgical, 94.8 (18.2) kg; Non‐surgical, 100.2 (20.9) kg	3 months: surgical, −7.68 (10.32) kg (−7.62%); non‐surgical, −8.42 (9.69) kg (−7.65%)	3 months: surgical, −7.62%; non‐surgical, −7.65%	5–9.9%: 21.1% (surgical), 20.8% (non‐surgical) at 3 months
Schwartz et al.^52^		111.1 (25.4) kg	−6.3 kg at 90 days	−12.8% at 90 days	
Toth et al.^53^	Surgical; 21–30 years	NR		Median −7.7% at nadir weight	
Shibuya et al.^47^	T2DM^b^	NR	3 months: T2DM, −3.30 (7.49) kg; non‐T2DM, −3.99 (5.67) kg (*p* = 0.21 vs. T2DM)		
**PHENTERMINE/TOPIRAMATE: RETROSPECTIVE COHORTS**					
Costello et al.^26^	General obesity	Median (range), 115.5 (75.8 to 134.5) kg	−2.2 kg (−16.8 to 0.1 kg) at 12 weeks	−2.8% at 12 weeks	
Grabarczyk^33^		124.7 (29.5) kg		−3.4 (14.2)% at 12 weeks; −4.1 (12.6)% at ≥ 20 weeks; −4.3 (14.1)% at 36 weeks	≥ 5%: 12 weeks, 34.1%; ≥ 20 weeks, 40.3%
Neoh et al.^42^		122.5 (29.7) kg (post‐VLED, pre‐drug)	−7.4 kg at nadir weight (*p* = 0.01 vs. post‐VLED, pre‐drug) −4.8 kg at final weight (*p* = 0.01 vs. post‐VLED, pre‐drug)		
Shibuya et al.^47^		104.1 (22.8) kg	−3.76 (5.6) kg at 12 weeks (*p* < 0.0001 vs. baseline)	−3.63% at 12 weeks	≥ 5% loss: 12 weeks, 36.7%
Schwartz et al.^52^	Surgical	105.8 (22.8) kg	−3.8 kg (−12.9%) at 90 days	−12.9% at 90 days	
Shibuya et al.^47^	T2DM^b^	NR	3 months: T2DM, −3.40 (5.72) kg; non‐T2DM, −3.88 (5.23) kg (*p* = 0.72 vs. T2DM)		
**LIRAGLUTIDE: PROSPECTIVE COHORTS**					
Suliman et al.^19^	General obesity	97.9 kg	−6.0 kg (95% CI 2.4, 9.4) at ≥ 16 weeks; −7.4 kg (−3.5, −11.0) at ≥ 28 weeks	−6.4% at ≥ 16 weeks; −7.6% at ≥ 28 weeks	
Suliman et al.^19^	Surgical	NR		≥ 16 weeks: surgical, −6.4%; non‐surgical, −6.1% (*p* < 0.0001 vs. baseline both groups) RYGB vs. SG at ≥ 28 weeks, −5.6% vs. − 3.3% (*p* = 0.025)	
Wharton et al.^20^		NR	7.6 months (mean follow‐up): all patients, −6.3 (7.7) kg; RYGB, −7.1 (8.7) kg; gastric band, −6.0 (7.2) kg; SG, −4.5 (4.5) kg (*p* < 0.05 vs. baseline, all groups)	7.6 months (mean follow‐up): all patients, −5.5%; RYGB, −6.6%; gastric band, −4.9%; SG, −3.6%	
**LIRAGLUTIDE: RETROSPECTIVE COHORTS**					
Gorgojo‐Martínez et al.^32^	General obesity	105.1 (18.6) kg	−6.4 kg (95% CI −7.5, −5.3) at 3–6 months; −7.7 kg (−9.0, −6.3) at end of follow‐up (*p* < 0.0001 vs. baseline, both time points)		> 5%: 3–6 months, 56.5%; end of follow‐up, 64.7%
Wharton et al.^48^		All, 115.5 (28.4) kg; ≥ 6 months persistent, 117.6 (31) kg; ≥ 4 months persistent, 115.9 (28.8) kg	6 months: all patients, −7.3 kg; ≥ 6 months persistent, −8.0 kg; ≥ 4 months persistent, −7.0 kg (*p* < 0.001 vs. baseline, all groups)	6 months: all patients, −6.5%; ≥ 6 months persistent, −7.1%; ≥ 4 months persistent, −6.3%	≥ 5%: 58.6% (all patients), 64.1% ( ≥ 6 months persistent), and 63.1% ( ≥ 4 months persistent) at 6 months
Rye et al.^51^	Surgical	NR		Median −7.1% (IQR, −5.1 to −12.2%) at 16 weeks; −9.7% (−7.8 to −13.9%) at 28 weeks	
Rye et al.^51^	Surgical, T2DM (*n* = 5)^b^	NR		Median −5.7% (IQR, −4.9 to −8.5%) at 16 weeks; −8.4% (−7.7 to −9.1%) at 28 weeks	
**NALTREXONE/BUPROPION: RETROSPECTIVE COHORTS**					
Shibuya et al.^47^	General obesity	110.5 (26.6) kg	−2.9 (5.5) kg (−2.66%) at 12 weeks (*p* < 0.0001 vs. baseline)	−2.66% at 12 weeks	≥ 5%: 12 weeks, 29.2%
Shibuya et al.^47^	T2DM^b^	NR	3 months: T2DM, −4.79 (4.92) kg; non‐T2DM, −2.16 (4.76) kg (*p* = 0.05 vs. T2DM)		
**POOLED AOMs: RETROSPECTIVE COHORTS**					
Nor Hanipah et al.^50^	Surgical Pooled: PHEN, PHEN/TPM, LORC, NTX/BPN	RYBG, 100.5 kg; LAGB, 106.5 kg; SG, 101.2 kg	3 months: all patients, −3.2 kg; RYGB, −3.2 kg; SG, −2.4 kg; LAGB, −4.5 kg 12 months: all patients, −2.4 kg; RYGB, −3.2 kg; SG, −0.3 kg; LAGB, −4.6 kg	3 months: all patients, −3.2%; RYGB, −3.2%; SG, −2.5%; LAGB, −4.6% 12 months: all patients, −2.2%; RYGB, −2.8% (*p* = 0.02 vs. SG); SG, −0.3%; LAGB, −4.6% (*p* = 0.01 vs. SG)	> 5%, 3 months: 30.0% (all patients), 31.1% (RYGB), 20.8% (SG), and 42.1% (LAGB) > 5%, 12 months: 36.9% (all patients), 40.0% (RYGB; *p* = 0.03 vs. SG), 21.4% (SG), and 58.8% (LAGB; *p* = 0.005 vs. SG)

Abbreviations: AOM, anti‐obesity medication; BPN, bupropion; CI, confidence interval; CVD, cardiovascular disease; DM, diabetes mellitus; IQR, interquartile range; LAGB, laparoscopic‐adjustable gastric band; LIRA, liraglutide; LORC, lorcaserin; NR, not reported; NS, not statistically significant; NTX, naltrexone; ORL, orlistat; PHEN, phentermine; RYGB, Roux‐en‐Y gastric bypass; SG, sleeve gastrectomy; T2DM, type 2 diabetes mellitus; TPM, topiramate; VLED, very‐low energy diet; WL, weight loss.

^a^ All comparisons are versus baseline (pre‐drug) and values are mean (SD) unless otherwise stated.

^b^ Subgroup of the overall study population; CVD subgroup not explicitly described, but assumed to include patients with cerebrovascular disease, coronary heart disease, and hypertension.

^c^ Short‐term use defined as phentermine for < 112 days and no subsequent use (referent group); medium‐term continuous use defined as phentermine for > 112 days up to 365 days, but no subsequent use; long‐term use defined as phentermine for > 112 days and > 365 days. Patients could move between categories (i.e., a medium‐term continuous user could become a long‐term user over time).

^d^ Including five eligible for this review (PHEN, NTX/BPN, ORL, LIRA, and PHEN/TPM).

#### 3.2.1 General obesity population

Across all studies in a general obesity population, AOMs were associated with a reduction in weight regardless of study design and duration. However, the magnitude of weight loss varied considerably from study to study (Table 3). With respect to achieving a clinically meaningful weight loss, the range of patients who lost ≥ 5% of their total body weight was 22.2% in a 12‐week orlistat study,^23^ up to 50% in a 12‐week phentermine study,^26^ and > 50% in two liraglutide studies (Table 3).^32^, ^48^

Few studies were identified that directly compared different AOMs of interest. Findings from these comparative analyses are summarized in Table 4. Orlistat was associated with a significantly poorer weight‐loss response compared with liraglutide after 3–6 months (*p*  <  0.0001)^32^ and a numerically lower absolute weight reduction compared with phentermine and PHEN/TPM at ≥ 20 weeks in a general obesity population.^33^ Patients receiving phentermine or PHEN/TPM were more than 50% more likely to experience a  ≥  5% weight loss compared with those receiving orlistat (*p*  <  0.01).^33^ In addition, in a matched cohort study, both phentermine and PHEN/TPM were associated with a greater weight reduction compared with NTX/BPN.^47^

**TABLE 4:** Weight outcomes (body weight change and categorical body weight loss) in active comparator studies

Study	Study population Drugs compared	Weight loss, kg and/or %	Categorical weight loss
Gorgojo‐Martínez, et al.^32^	General obesity LIRA vs. ORL	Crude mean difference: −2.53 kg (95% CI −3.67, −1.4) at 3–6 months (*p* < 0.001) −4.37 kg (95% CI −5.98, −2.76) at last study visit (*p* < 0.0001)	OR (95% CI) for ≥ 5% loss, LIRA vs. ORL: 3–6 months, 3.38 (1.90, 6.04); last study visit, 7.06 (3.81, 13.07) (*p* < 0.0001 both time points)
Grabarczyk^33^	General obesity ORL, PHEN, PHEN/TPM, LORC	At ≥ 20 weeks: ORL, −2.1%; PHEN, −3.6%; PHEN/TPM, −4.1%; LORC, −3.6% (*p* = NS between groups)	≥ 5% loss at ≥ 20 weeks: ORL, 27.1%; PHEN, 38.5%; PHEN/TPM, 40.3%; LORC, 34.6% (*p* = NS between groups) OR (95% CI) for ≥ 5% loss at ≥ 20 weeks: LORC vs. ORL, 1.27 (0.98, 1.65); PHEN vs. ORL, 1.55 (1.20, 2.00) (*p* < 0.01); PHEN/TPM vs. ORL, 1.59 (1.19, 2.10) (*p* < 0.01)
Li et al.^41^	General obesity PHEN vs. PHEN/FEN	NS difference in men and women at 8 and 12 weeks Women at 4 weeks: PHEN, −3.6 kg vs. PHEN/FEN, −4.1 kg (*p* = 0.042)	
Shibuya et al.^47^	General obesity PHEN, PHEN/TPM, LORC, NTX/BPN	Weight loss difference at 12 weeks (PSM cohorts): PHEN vs. PHEN/TPM, 0.14 (8.51) kg (*p* = NS); PHEN vs. LORC, 1.46 (10.95) kg (*p* = 0.013); PHEN vs. NTX/BPN, 1.14 (8.36) kg (*p* = 0.030) PHEN/TPM vs. LORC, 1.48 (11.47) kg (*p* = 0.026); PHEN/TPM vs. NTX/BPN, 0.77 (7.51) kg (*p* = NS) LORC vs. NTX/BPN, 0.64 (11.96) kg (*p* = NS)	
Elhag et al.^49^	Surgical and non‐surgical PHEN vs. LORC	Non‐surgical at 3 months PHEN, −8.42 (−9.69) kg (−7.65 [8.26]%); *p* = 0.003 vs. LORC LORC, −2.98 (−4.15) kg (−2.99 [3.72]%) Surgical at 3 months PHEN, −7.68 (−10.32) kg (−7.62 [9.80]%); *p* = 0.012 vs. LORC LORC, −1.81 (−4.54) kg (−1.86 [5.06]%)	5–9.99% loss at 3 months, PHEN vs. LORC: non‐surgical, 20.8% vs. 8.6%; surgical, 21.1% vs. 25.9%
Schwartz et al.^52^	Surgical PHEN vs. PHEN/TPM	Weight difference at 90 days: −1.35 kg (95% CI 0.17, 2.53); *p* = 0.025	

*Note*: Only studies that included a comparison with a drug of interest are listed here.

Abbreviations: AOM, anti‐obesity medication; BPN, bupropion; CI, confidence interval; FEN, fenfluramine; LIRA, liraglutide; LORC, lorcaserin; NS, not statistically significant; NTX, naltrexone; OR, odds ratio; ORL, orlistat; PHEN, phentermine; PSM, propensity score matched; TPM, topiramate.

#### 3.2.2 Diabetes population

Among the five studies that evaluated weight response among patients with obesity and T2DM, treatment with orlistat, phentermine, PHEN/TPM, and NTX/BPN was associated with a reduction in weight that appeared to be comparable with losses observed in a general population of individuals with obesity (Table 3).^16^, ^24^, ^30^, ^34^, ^47^ One study demonstrated that in patients with T2DM, orlistat in combination with participation in a clinical weight‐loss program resulted in a numerically better weight‐loss response compared with orlistat alone, although the difference failed to reach statistical significance (Table 3).^34^ In another study, no differences were reported in weight loss between patients with or without T2DM treated with phentermine, PHEN/TPM, or liraglutide, but a difference was demonstrated with NTX/BPN (T2DM, −4.8 kg vs. non‐T2DM, −2.2 kg; *p* = 0.05; Table 3) although patient numbers were low (*n* = 32).^47^

#### 3.2.3 Postsurgical population

In patients with obesity who had previously undergone bariatric surgery and experienced subsequent weight regain or insufficient weight loss postoperatively, treatment with phentermine, PHEN/TPM, and liraglutide all resulted in weight reduction (Table 3).^19^, ^20^, ^49^, ^50^, ^51^, ^52^, ^53^ Weight loss was experienced across studies regardless of patient population (e.g., T2DM or young adults^51^, ^53^) or surgery type. Furthermore, in one study, there was no apparent difference in percentage total weight loss with phentermine between surgical and non‐surgical patients, and in another study, no difference was observed between surgical and non‐surgical cohorts treated with liraglutide with respect to weight loss from baseline prior to drug initiation.^19^, ^49^ Significantly greater weight reductions were, however, reported in a Canadian prospective cohort study among liraglutide‐treated patients who had undergone RYGB compared with SG (Table 3).^20^ Similarly, in one study that pooled data from multiple AOMs, weight loss was greater in patients who received drug treatment following RYGB compared with those treated post‐SG.^50^

Very few comparative data were available in surgical patients, but one study provided evidence that phentermine may produce superior weight loss compared with PHEN/TPM in surgical patients, although it should be noted that the number of patients receiving PHEN/TPM in this analysis was small (*n* = 6) (Table 4).^52^

### 3.3 Cardiometabolic risk factors

Cardiometabolic risk factors were less well studied among the included articles. Thirteen studies overall (around 30% of those included; general obesity population [with or without DM], *n* = 12; surgical population, *n* = 1) evaluated the impact of AOMs on parameters including blood pressure, heart rate (HR), lipids, fasting blood glucose, and glycated hemoglobin (HbA_1c_). The effects of orlistat and phentermine were evaluated most frequently, followed by liraglutide and PHEN/TPM. Results varied across studies, with some demonstrating a positive impact on cardiometabolic risk factors and others showing no effect. An overview of the trends across studies is shown in Table 5.

**TABLE 5:** Cardiometabolic risk factors affected by AOM therapy in real‐world practice

Study	Study population	Study duration, months	SBP	DBP	HR	TG	TC	LDL‐C	HDL‐C	FBG	HbA_1c_
**ORLISTAT**											
Ahn et al.^23^	General obesity	6	**↓** *	**↓** *		**↓**	**↓** *	**↓**	**↔**	**↓** *	
Gorgojo‐Martínez et al.^32^		3–6	**↓** *	**↔**	**↔**	**↓**		**↓** *	**↓** *	**↓** *	
Grabarcyzk^33^		6	**↔**	**↔**		**↓**		**↓**	**↔**		**↔**
Wirth^18^		6–9	**↓**	**↓**	**↔**	**↓** *	**↓** *	**↓** *	**↑** *	**↓**	
Allie et al.^24^	DM^b^	3–6					**↓** *	**↓** *	**↓** *		**↓**
Gorgojo‐Martínez et al.^32^ ^a^		3–6								**↓** *	**↓** *
Graham et al.^34^		6	**↓**	**↔**			**↓** *	**↔**	**↓** ^c^		**↓** * ^c^
Rowe et al.^16^		6									**↓** *
Wirth^18^ ^d^		6–9								**↓** *	
Wirth^18^ ^d^	Dyslipidemia	6–9				**↓** *	**↓** *	**↓** *	**↑** *		
Wirth^18^ ^d^	Hypertension	6–9	**↓** *	**↓** *							
**PHENTERMINE**											
Grabarczyk^33^	General obesity	6	**↔**	**↔**		**↓**		**↔**	**↔**		**↔**
Hendricks et al.^36^ ^e^		12–24	**↓** *	**↓** *	**↑** ^e^						
Kim et al.^15^		3	**↓**	**↓**							
Lewis et al.^40^ ^f^		12–24	**↓** *	**↔**	**↑**						
Elhag et al.^49^	Surgical	3				**↔**	**↔**	**↔**	**↔**	**↔**	**↔**
**PHENTERMINE/TOPIRAMATE**											
Grabarczyk^33^	General obesity	6	**↔**	**↔**		**↓**		**↓**	**↔**		**↔**
Neoh et al.^42^		To nadir weight	**↓** *	**↔**							
**LIRAGLUTIDE**											
Gorgojo‐Martínez et al.^32^	General obesity	3–6	**↓**	**↓** *	**↑** *	**↓** *		**↓** *	**↓**	**↓** *	**↓** *
Wharton et al.^48^		6	**↓** *	**↔**							**↓** *

Abbreviations: DBP, diastolic blood pressure; FBG, fasting blood glucose; HbA_1c_, glycated hemoglobin; HDL‐C, high‐density lipoprotein cholesterol; HR, heart rate; LDL‐C, low‐density lipoprotein cholesterol; SBP, systolic blood pressure; TC, total cholesterol; TG, triglycerides.

*Note*: ↑ and ↓ indicates increase or decrease vs. baseline (pre‐drug) levels; ↔ indicates no change vs. baseline (pre‐drug) values.

^a^ Subanalysis including the 20.3% of patients with obesity and T2DM.

^b^ Specifically T2DM in Allie et al.,^24^ Gorgojo‐Martínez et al.,^32^ and Graham et al.^34^; 91% T2DM in Rowe et al.^16^; DM type not specified in Wirth.^18^

^c^ HDL‐C reduction in ORL‐only group, not ORL + WL clinic; significant HbA_1c_ reduction only in ORL + WL clinic (*p* = NS ORL alone).

^d^ Significant reduction from baseline in parameters in patients with and without DM, dyslipidemia, and hypertension, but reduction was greater in subgroups with comorbidities.

^e^ Phentermine‐treated patients also enrolled in a highly prescriptive weight‐management program; increase in HR phentermine‐treated patients only versus decrease in WL program only group.

^f^ Data from medium and long‐term continuous users of phentermine.

* Statistically significant within‐arm increase or decrease vs. baseline (pre‐drug) values.

#### 3.3.1 Orlistat

In the six studies that evaluated the effect of orlistat on blood pressure, systolic blood pressure (SBP) was significantly reduced in three studies and numerically reduced or unchanged in three, while no effect or a numerical decrease in diastolic blood pressure (DBP) was observed in four studies and a significant reduction reported in two (Table 5). Triglycerides (TG), total cholesterol (TC), and low‐density lipoprotein cholesterol (LDL‐C) were generally reduced in association with orlistat treatment, while impact on high‐density lipoprotein cholesterol (HDL‐C) was more variable (Table 5). Glycemic parameters were consistently reduced in patients with obesity and DM (mostly T2DM) who received orlistat.^16^, ^18^, ^24^, ^32^, ^34^ One German postmarketing study also evaluated the effects of orlistat on cardiometabolic risk factors in subgroups of patients with comorbidities and demonstrated that improvements in blood pressure or lipid parameters were greater in individuals with hypertension or dyslipidemia, respectively.^18^

In a single comparative study, no clinically significant differences from baseline to 6 months in blood pressure, lipids, or HbA_1c_ was observed between patients treated with orlistat, phentermine, or PHEN/TPM.^33^

#### 3.3.2 Phentermine and PHEN/TPM

Like orlistat, phentermine appeared to be associated with a reduction in SBP (Table 5). However, small increases in HR from baseline were reported in phentermine‐treated patients, although this did not reach statistical significance.^36^, ^40^ Two studies reported on the effect of PHEN/TPM on cardiometabolic risk factors, with few changes in blood pressure, lipids, or glycemia observed (Table 5).^33^, ^42^

#### 3.3.3 Liraglutide

Few data (*n* = 2 studies) were identified regarding the impact of liraglutide on cardiometabolic risk factors (Table 5). Where studied, liraglutide was generally associated with a reduction in blood pressure, lipids, and glycemic parameters.^32^, ^48^

#### 3.3.4 Postsurgical patients

No changes in lipid or glycemic parameters were reported in a single study including patients who received phentermine for weight gain/insufficient weight loss after bariatric surgery (Table 5).^49^

### 3.4 Existing comorbidities

Five of the identified studies in a general obesity population (with or without T2DM) also evaluated the impact of AOMs on existing comorbidities, which was generally reported as a change in specific medications. For example, antihypertensive, glucose‐lowering, and lipid‐lowering drug use was reported to be reduced following orlistat initiation in three studies including patients with obesity and comorbid diseases,^16^, ^18^, ^27^ but another study in patients with T2DM failed to demonstrate any significant changes.^24^ Resolution of baseline prediabetes occurred in fewer orlistat‐treated patients with obesity compared with liraglutide‐treated patients in a Spanish observational study and more patients progressed to T2DM (6.1% vs. 0%; *p*  <  0.0001).^32^

#### 3.4.1 Postsurgical patients

Both phentermine and PHEN/TPM failed to have an impact on comorbid hypertension and DM in post‐bariatric surgery patients.^52^

### 3.5 Adverse events

Data on the incidence of AEs in AOM‐treated patients were reported in 24 studies overall (57% of included) and the level of detail in the data varied considerably between investigations. Of these studies, 22 were in general obesity populations (with or without DM), two pooled AE data across surgical and nonsurgical patient cohorts,^19^, ^49^ and two specifically reported AE data in postsurgical patients.^20^, ^51^ Overall, AE data were reported most frequently for orlistat (*n* = 12 studies) and phentermine (*n* = 9), with only four and two studies providing information regarding liraglutide or PHEN/TPM, respectively. No AE data were identified for NTX/BPN. Where reported, AEs appeared to be mild to moderate in severity and were mostly short‐lived.

#### 3.5.1 Orlistat

In orlistat studies, AEs affecting the gastrointestinal system were the most commonly reported events.^17^, ^18^, ^21^, ^22^, ^23^, ^32^, ^34^, ^44^, ^45^ Acute liver injury and colorectal cancer were specifically evaluated in orlistat‐treated patients included in the UK Clinical Practice Research Database (CPRD) and no increase in the incidence of either event was observed.^29^, ^37^

#### 3.5.2 Phentermine and PHEN/TPM

A range of different AEs was reported in phentermine‐treated patients, with palpitations, dry mouth, insomnia, constipation, fatigue, and dizziness being among the most common.^15^, ^36^, ^41^, ^49^ Cardiovascular (CV) and cerebrovascular events were specifically evaluated in four studies. In two nested case–control studies that used data from the UK CPRD, no increase in the incidence of stroke or cardiac‐valve abnormalities was observed in patients treated with phentermine.^28^, ^39^ In a US retrospective cohort study that used data from electronic medical records, it was demonstrated that there was no increase in the risk of CV disease or death with phentermine use for up to 3 years after initiation.^40^ Another US study that utilized claims data also found that there was no increase in the risk of major adverse CV events (MACE; hospitalization for acute myocardial infarction [MI], stroke, or in‐hospital CV death) in phentermine users.^46^ Similarly, this study also demonstrated no increase in the risk of MACE in current users of PHEN/TPM. In one other study that provided data for PHEN/TPM‐treated patients, it was reported that paresthesia, cognitive changes, dry mouth, and headache were the most common AEs.^42^

#### 3.5.3 Liraglutide

AEs associated with liraglutide were reported in two studies in a general obesity population.^19^, ^32^ The most common AEs in liraglutide‐treated patients were mostly gastrointestinal in nature, including nausea and vomiting, and diarrhea.

#### 3.5.4 Postsurgical patients

Two studies evaluated AEs associated with liraglutide in patients who had previously undergone bariatric surgery. Among the most commonly reported AEs were nausea, headache, constipation, and diarrhea.^20^, ^51^

### 3.6 Adherence, persistence, and discontinuation

Compliance outcomes comprising adherence, persistence, and discontinuation were reported in 21 studies (50% of included records): general obesity population (*n* = 18), pooled surgical and nonsurgical cohorts (*n* = 1), and postsurgical (*n* = 2). Overall, orlistat was the most frequently evaluated AOM in this regard (*n* = 14 studies), followed by liraglutide (*n* = 5), phentermine (*n* = 4), PHEN/TPM (*n* = 3), and NTX/BPN (*n* = 1). The main findings with respect to these outcomes are summarized in Table 6. Across studies, adherence, persistence, and discontinuation were measured in multiple different ways, rendering it impossible to compare outcomes from one investigation to the other. However, there was a general pattern of poor compliance with all AOMs. For example, in a US retrospective observational cohort study using data from the Veterans Affairs Corporate Data Warehouse that used the medication possession ratio (MPR) to determine 6‐month adherence, the highest rate reported was only 38.2% in PHEN/TPM‐treated patients, with other AOMs performing even more poorly (Table 6).^33^ Low adherence was similarly reported in another US study that used proportion of days covered as the metric.^31^ This study also provided estimates of persistence and demonstrated that only 18.1%, 27.3%, and 41.8% of patients treated with NTX/BPN, PHEN/TPM, or liraglutide, respectively, were persistent at 6 months.^31^ High proportions of patients discontinued treatment within 6–12 months (Table 6); reported reasons included AEs^15^, ^17^, ^19^ and perceived lack of weight‐loss effectiveness.^22^, ^44^

**TABLE 6:** Adherence, persistence, and discontinuation of AOMs

Study	Study population	Adherence	Persistence	Discontinuations	AOM comparisons
**ORLISTAT: PROSPECTIVE**					
Hollywood and Ogden^14^	General obesity	Self‐rated total adherence, 30.4%		47.5% discontinued by 6 months	
Schwartz et al.^17^			ORL used for median 90% of days since study enrollment	8.5% discontinued due to AEs	
Wirth^18^		Physician‐rated compliance: excellent, 21.3%; good/very good, 61.8%; moderate, 10.7%; inadequate, 5.1%; missing, 1.1%^a^	Mean duration use, 7.1 months		
**ORLISTAT: RETROSPECTIVE**					
Acharya et al.^22^; Perrio et al.^44^	General obesity			30.3% discontinued in first 3 months; 68.9% by study end	
Beermann et al.^25^		Complete adherence to approved indication, 6.5%^b^			
Gorgojo‐Martínez et al.^32^			Persistence: 3–6 months, 64.8%; 12 months, 46.8%; end of follow‐up, 19.5% Interrupted therapy at least once for ≥ 7 days and restarted within follow‐up period, 35.8%		Persistence: ORL < LIRA
Grabarczyk^33^		6‐month MPR ≥ 80%, 17.5%; MPR, 0.50 (0.26)			Adherence: ORL < PHEN/TPM
Hemo et al.^35^			Persistence: ≥ 4 months, 15.5% Average duration of therapy, 2.1 months		Persistence: SIB > ORL (*p* < 0.001)
Padwal et al.^43^			Persistence: 6 months, 18%; 1 year, 6%; 2 years, 2%		
Allie et al.^24^	T2DM			44% discontinued within 3 months of initiation	
Graham et al.^34^		ORL vs. ORL + WL program: no difference in adherence (*p* = 0.865)^c^		Discontinued within 6 months: 34% (all patients); 17% (ORL); 61% (ORL + WL program) (*p* = 0.004 vs. ORL alone)	
Rowe et al.^16^	DM (91% T2DM)			18% discontinued within 6 months	
Horie et al.^38^	≥ 60 years		Mean (SD) duration of therapy, 8.7 (5.0) months		
**PHENTERMINE: PROSPECTIVE**					
Kim et al.^15^	General obesity	Good compliance in 26.3%^d^	62% completed 12‐week treatment	9.0% discontinued within 12 weeks due to AEs	
**PHENTERMINE: RETROSPECTIVE**					
Grabarczyk^33^	General obesity	6‐month MPR ≥ 80%, 29.4%; MPR, 0.57 (0.29)			
Li et al.^41^				8.3% of male patients discontinued by week 8 3.9% of female patients discontinued by week 4; 7.8% by week 8 No further discontinuations by week 12	
Schwartz et al.^52^	Surgical			No discontinuations due to hypertension, cardiac arrythmias, or insomnia; one discontinuation each due to headaches and nausea	
**PHENTERMINE/TOPIRAMATE: RETROSPECTIVE**					
Grabarczyk^33^	General obesity	6‐month MPR ≥ 80%, 38.2%; MPR, 0.65 (0.26)			Adherence: PHEN/TPM > ORL (*p* < 0.05)
Ganguly et al.^31^		6‐month PDC ≥ 80%, 20.6%; PDC, 0.47 (0.29)	Persistence: 3 months, 49.0%; 6 months, 27.3%; 9 months, 16.8%; 12 months, 10.9% ≥ 1 prescription refill beyond index claim, 72% Switch to alternative AOM, 13.7% (to LORC, 27.3%; to LIRA, 28.0%; to NTX/BPN, 44.8%)		Persistence: PHEN/TPM < LIRA Discontinuation: PHEN/TPM > LIRA
Schwartz et al.^52^	Surgical			No discontinuations due to hypertension, cardiac arrhythmias, or insomnia	
**LIRAGLUTIDE: PROSPECTIVE**					
Suliman et al.^19^	General obesity			20% discontinued after median 108 days; 6.7% of study population due to AEs	
Wharton et al.^20^	Surgical		Persistence: 36.8% at 1 year	23.9% discontinued by 1 year	
**LIRAGLUTIDE: RETROSPECTIVE**					
Ganguly et al.^31^	General obesity	6‐month PDC ≥ 0.80, 27.4%; PDC, 0.56 (0.28)	Persistence: 3 months, 62.6%; 6 months, 41.8%; 9 months, 33.0%; 12 months, 28.2% Switching in first 6 months, 3.7% (to LORC, 21.7%; to NTX/BPN, 54.6%; to PHEN/TPM, 23.7%)		Persistence: LIRA > LORC, PHEN/TPM, NTX/BPN at 6 and 12 months (*p* < 0.001) Discontinuation: LIRA < LORC (HR, 0.46), NTX/BPN (HR, 0.48), PHEN/TPM (HR, 0.64) (*p* < 0.0001)
Gorgojo‐Martínez et al.^32^			Persistence: 3–6 months, 75%; 12 months, 61%; end of follow‐up, 55% Therapy interruption at least once for ≥ 7 days, with restart within follow‐up, 11%		Persistence: LIRA > ORL at 3–6 months (*p* = 0.052), 12 months (*p* = 0.011), and end of follow‐up (*p* < 0.0001) Therapy interruption: LIRA < ORL (*p* < 0.0001)
Wharton et al.^48^			Persistence: ≥ 4 months, 67.5%; ≥ 6 months, 53.7%	Discontinuations: ≥ 4‐month persistent cohort, 28.1%; ≥ 6‐month persistent cohort, 51.5%	
**NALTREXONE/BUPROPION: RETROSPECTIVE**					
Ganguly et al.^31^	General obesity	6‐month PDC ≥ 80%, 11.1%; PDC, 0.38 (0.26)	Persistence: 3 months, 34.2%; 6 months, 18.1%; 9 months, 12.7%; 12 months, 9.2% Switching to alternative AOM, 6.9% (to LIRA, 40.7%; LORC, 35.1%; PHEN/TPM, 24.2%)		Persistence: NTX/BPN < LIRA Discontinuation: NTX/BPN > LIRA

Abbreviations: AE, adverse event; AOM, anti‐obesity medication; BMI, body mass index; BPN, bupropion; DM, diabetes mellitus; LIRA, liraglutide; LORC, lorcaserin; MPR, medication possession ratio; NTX, naltrexone; ORL, orlistat; PDC, proportion of days covered; PHEN, phentermine; SD, standard deviation; SIB, sibutramine; T2DM, type 2 diabetes mellitus; TPM, topiramate; WL, weight loss.

^a^ Rated on a 5‐point scale; unclear from the publication whether compliance just relates to medication adherence or to all aspects of management, including dietary restrictions.

^b^ Complete adherence to approved indication characterized as having correct BMI at initiation, approved weight reduction during the pre‐drug diet period, and continued orlistat treatment after 3 months only with an approved weight reduction of ≥ 5%.

^c^ Adherence for the ORL + WL program group was defined as patients taking 120–360 mg/d, as the number of pills per day may vary with number of meals consumed. Thus, if ≥ 1 dose was ingested daily, the patient was considered adherent. Adherence for ORL‐only patients was defined as taking ≥ 80% of their weekly dose or having computerized prescription records indicating sufficient medication supply between visits.

^d^ Compliance rate was measured according to the percentage of patients who took medication during the study period; if the rate was > 80%, compliance was considered good and if < 80%, it was considered poor.

#### 3.6.1 Comparative studies

A direct comparison between different AOMs was undertaken in four retrospective studies (Table 6).^31^, ^32^, ^33^, ^35^ In one study, more patients remained on liraglutide at 12 months versus orlistat (*p* = 0.011) and at the end of follow‐up persistence was higher (55% vs. 19.5%; *p*  <  0.0001).^23^ However, after adjustment for baseline factors, there was no significant difference between the persistence curves. A significantly lower risk of discontinuation with liraglutide was demonstrated in another study compared with PHEN/TPM and NTX/BPN after adjustment for baseline factors.^31^, ^32^ Conversely, where studied, adherence and persistence were generally worse with orlistat compared with liraglutide (unadjusted analysis), PHEN/TPM, and phentermine.^32^, ^33^, ^35^

#### 3.6.2 Postsurgical patients

Limited data were identified regarding compliance outcomes in postsurgical patients.^20^, ^52^ In one study, discontinuation was observed in 24% of patients treated with liraglutide within 1 year, with the most common reasons being lack of weight loss efficacy, cost, and AEs.^20^

## 4 DISCUSSION

The current review identified numerous studies that provide evidence for the effectiveness and tolerability of AOMs in real‐world practice and describe an experience more typical of patients who are seeking weight‐loss solutions from their healthcare professional. Although there was a wide disparity in designs, patient populations, and durations across studies that challenged the drawing of definitive conclusions, it was clear that available AOMs were associated with a reduction in weight from baseline in a general obesity population, and these data do appear to support the efficacy of AOMs previously reported in tightly controlled RCTs.^11^ Limited evidence from the included studies also suggests that in a general obesity population, AOMs may be accompanied by positive changes in other cardiometabolic risk factors that could be indicative of downstream improvements in existing obesity‐related comorbidities such as T2DM, dyslipidemia, and hypertension. However, few studies evaluated these outcomes and those that did generally used a reduction in drug use for specific conditions as a surrogate and were of insufficient duration to robustly capture significant improvements. Furthermore, comorbidities beyond the usual cardiometabolic risk factors of blood pressure, lipids, and glycemic parameters were not assessed. Other comorbidities known to have strong associations with obesity, such as non‐alcoholic steatohepatitis, obstructive sleep apnea, arthritis, depression, and cancer, were not represented.

Some of the data from patient subpopulations warrants further discussion. In contrast to RCTs, which have consistently shown less weight loss with AOMs in populations with T2DM compared to those without T2DM, the few real‐world studies that evaluated these drugs in people with T2DM suggest comparable effectiveness.^54^ One study even demonstrated better response with NTX/BPN in people with T2DM versus those without T2DM, although the statistical significance was borderline. On one hand, it is likely that these real‐world studies do not adequately control for unmeasured confounding factors; for example, it is possible that people with T2DM selected for treatment with NTX/BPN had a more hedonic phenotype (e.g., reward‐based eating behaviors) that could have made this cohort more responsive to this treatment compared with people without T2DM. The included studies also did not test the relative effectiveness of different agents in people with T2DM and, therefore, conclusions cannot be drawn on whether a particular AOM, such as NTX/BPN, is more effective than current preferred agents (e.g., glucagon‐like peptide‐1 receptor agonists) in this population. On the other hand, the demonstrable effectiveness in these real‐world studies confirms the feasibility of weight loss success with AOMs generally in people with T2DM despite the known greater resistance to weight loss in this population.^54^

Weight regain or inadequate weight loss after bariatric surgery is challenging to manage since these patients have already undergone the gold‐standard treatment modality for obesity. Furthermore, these patients likely represent a distinct group of individuals with different background characteristics compared with the general obesity population. The RWE studies identified in this review indicate that AOMs may be a viable adjunctive treatment option for certain postsurgical patients.^19^, ^20^, ^49^, ^51^, ^52^, ^53^ Findings from real‐world practice also suggest that AOMs could have varying effects depending on type of metabolic surgery,^19^, ^20^, ^50^ which may be due to the interplay between different surgical procedures and drug mechanism of action. These studies were also mostly conducted in specialized weight management centers, underscoring that, at present, AOMs are not routinely prescribed after surgery except in obesity centers with expertise in managing such patients.

Taken together, the data across studies suggest that AOMs are well tolerated in real‐world practice. AE severity was infrequently reported, but where details were given, it was generally noted that AEs appeared to be mild to moderate in severity and were mostly short‐lived. However, in some studies, AEs constituted one of the main reasons for discontinuation. Although the reason for this disconnect is not completely clear and the severity of AEs leading to discontinuation is not specified in the included studies, it is possible that healthcare providers and/or patients have a lower threshold for tolerating AEs in the real world compared to clinical trials. Historically, the medical management of obesity has been perceived as an elective option, providers have not received formal training in obesity management, and those who prescribe AOMs have faced judgment and stigma from peers. Collectively, these factors may lower provider tolerance for AEs or limit their confidence in managing mild to moderate AEs in clinical practice. Patients in the real world may also present with more multi‐morbidity and clinical complexity compared with those in clinical trials, and that too may lower the tolerance for mild to moderate AEs and lead to more discontinuation of AOMs.

Adherence and persistence are important determinants of AOM effectiveness, and it is known that weight loss is not typically sustained upon cessation of therapy.^32^ Again, wide variations were observed in reporting methods and presentation of compliance results across studies, but generally, adherence and persistence with AOMs in clinical practice appeared to be poor. No conclusive evidence could be drawn regarding superiority of one AOM over another with respect to compliance due to the limited number of comparative studies identified. However, adherence and persistence with liraglutide appeared to be higher than orlistat in an unadjusted (but not adjusted) analysis in one study, and higher than NTX/BPN and PHEN/TPM in another.^31^, ^32^ There was also the suggestion that PHEN/TPM may be associated with better adherence and persistence versus other AOMs, except liraglutide.^31^, ^33^ Overall, the field would benefit from agreed standards to measure these compliance outcomes to allow for comparability across studies.

The reasons underlying poor compliance are likely to be multifactorial and could include variable weight‐loss efficacy or perceived ineffectiveness, intolerable AEs, drug costs, inadequate healthcare provider training, and lack of patient education.^19^, ^20^, ^22^, ^44^ Many patients may have unrealistic expectations regarding the extent of weight loss that can be achieved with their medication and become discouraged early in therapy if the results are not as dramatic as they hoped.^31^ Both providers and patients also tend to view AOMs as a jump start for weight loss rather than chronic therapy that extends to weight maintenance, and this may account for lack of persistence even in those who initially achieve meaningful weight loss. Since the benefits of short‐term weight loss are unclear, low compliance with AOMs raises important questions regarding the cost‐effectiveness or value of the treatment as it is currently applied in the real world. Low persistence and adherence will need to be addressed to sustain the observed real‐world effectiveness of AOMs and achieve the potential long‐term benefits of AOM‐induced weight loss.

Real‐world data are emerging as an important component of the overall evidence base for understanding the utility of medications across a range of patient populations.^12^ These data may represent a valuable supplement to those obtained in RCTs. For example, sibutramine was withdrawn from global markets due to CV safety concerns reported in an RCT.^55^ Real‐world studies failed to demonstrate such CV risks in a more generalizable patient population, suggesting that the marketing authorization for sibutramine may have been inappropriately withdrawn for patients without pre‐existing CV disease.^13^, ^56^, ^57^

More studies identified by the current search were conducted in secondary/tertiary care compared with primary care settings. However, as obesity rates continue to climb and its acknowledgment as a chronic disease continues to grow, more and more individuals will seek weight management advice from their primary care physician. Therefore, it is important to gain a better understanding of the experience of patients in this setting. The fact that 12 primary care studies were identified by this review suggests that AOMs are effective and well tolerated in this setting. These studies provide valuable information regarding the translation of obesity management from the specialist to the generalist setting and the feasibility of scaling the pharmacologic management of obesity.

One of the challenges with RWE is the difficulty in interpreting data across studies. Methods, populations, data collection, and reporting vary considerably from one evaluation to the next. In addition, confounding by background lifestyle measures is a major issue. Clinical guidelines for obesity management and label indications for FDA‐approved AOMs specify that pharmacotherapy for obesity only be used as an adjunct to lifestyle modification, where such modification is subject to varied interpretation. This requirement means that measurement of the true efficacy of an individual AOM alone is rarely, if ever, achieved. Certainly, a range of adjunct lifestyle measures—including calorie‐deficit diets, nutritional counseling, physical activity recommendations, and/or intensive behavioral therapy—have been adopted as part of the study design in RCTs evaluating AOMs. Since there is limited objective assessment of adherence to such recommendations in these trials, adjunct lifestyle measures can be viewed as a major source of confounding. Similarly, the findings from this review indicate that participants in real‐world studies may be enrolled in rigorous weight‐management programs or following specific dietary restrictions and programs of physical activity. However, the capture of this information is variable and adherence to such measures is seldom reported. This contributes to the heterogeneity among real‐world studies, making it difficult to summarize weight‐loss effects of a single drug, let alone compare effects across different medications. In the absence of appropriate control arms in real‐world studies, the interaction between AOM and lifestyle measures is unclear. In a similar way, it is difficult to appreciate the real‐world impact of AOMs in the studies including people with T2DM because few of these provided details of any concomitant glucose‐lowering medications. Since many of these agents also promote weight gain or weight loss, they could have an impact on AOM effectiveness in these patients that confounds the results.

The current review is subject to several limitations that relate to the search itself, the evidence base, and issues inherent in the methods of real‐world studies. While the search was conducted using a robust and reproducible protocol, the approach was largely pragmatic, and it cannot be ruled out that other studies relevant to the research question may have been published. The research question focused on specific FDA‐approved AOMs deemed to be relevant to the current pharmacologic management of obesity. As such, studies that provided RWE for the effectiveness of AOMs generally without specific drug‐level data were not a part of the search strategy. In addition, a two‐stage approach was adopted for the review of search results; at the first stage, the decision to include or exclude a publication is made based on review of the title/abstract and not on a comprehensive review of the full‐text of the article, so it is possible that potentially relevant studies are excluded at this stage due to lack of detail in the title or abstract. For example, if the AOMs of interest were not specifically mentioned by name in the title/abstract, the study did not meet our eligibility criteria, but it could be that the full‐text of the publication did provide disaggregated data on that agent. Furthermore, inconsistencies in the description of RWE in the literature, the range of terminologies used, and the lack of clarity in methods for data collection—even in the full‐text of some papers—made the decision to include challenging in some cases. The reviewers were also compelled to exercise a level of value judgement as to whether a study truly reflected real‐world practice. For example, some studies—though conducted in a clinical setting and termed observational—had strict inclusion/exclusion criteria, highly prescriptive scheduling and conduct of clinic visits, and did not appear to fully reflect patient behaviors were they not to have been included in the study. In such cases, the studies were extensively discussed among reviewers until consensus was met.

While the search was designed to identify a wide array of outcomes associated with AOMs, substantial gaps were evident in the RWE. Few studies were identified that reported data on outcomes other than weight change, AEs, and compliance. Data on cardiometabolic risk factors was generally limited, although this is perhaps not surprising given that healthcare providers may not routinely monitor metabolic labs in clinical practice due to the constraints of cost and insurance coverage. Only two studies each (5% of the total) were identified as including limited data on economic outcomes^16^, ^49^ or patient‐reported outcomes.^17^, ^18^ This is also expected since routine real‐world data sources like EMR or claims databases will often not capture patient‐reported outcomes. There is, therefore, a need for other forms of data capture in a real‐world setting (e.g., in the form of pragmatic trials) to evaluate these types of outcomes.

Another identified gap was that most studies were single arm, comparing the impact of each drug to baseline; practically none included a control arm, and relatively few studies were identified that directly compared different AOMs. Furthermore, few studies included details regarding the analytical approach for handling missing data. Since these methods can influence bias in the results, it is difficult to know if accurate conclusions about the data have been drawn in individual studies. Many of the included studies also contained small numbers of patients and were of short duration; data are, therefore, lacking on the maintenance of weight loss. Finally, selection bias is inherent in many of the studies since healthcare professionals often do not proactively address obesity with the use of AOMs, but rather patients seeking weight‐loss options beyond diet and exercise request medication from their physician or self‐refer to weight‐management specialists. It is not known from the included studies whether physicians provided objective advice and counseling regarding AOMs when medically indicated independent of patient requests. As such, the data may not represent the real‐world effectiveness of AOMs in the indicated population, but rather in a subset of patients who may have proactively requested this treatment option.

## 5 CONCLUSIONS

RWE for the effectiveness and safety of AOMs were identified in a diverse obesity population. Such evidence can supplement the findings previously reported in tightly controlled RCT patient samples. Across studies employing prospective and retrospective designs, AOMs were consistently demonstrated to reduce weight in a general population of patients with obesity/overweight, in patients who had regained weight or experienced inadequate weight loss after bariatric surgery, and in specific patient subgroups such as T2DM. Weight loss was often accompanied by positive changes in other cardiometabolic risk factors, when measured. Although AOMs were well tolerated in real‐world studies with mostly mild to moderate AEs, a general pattern of poor compliance was apparent with all treatments, the reasons for which will need to be better understood and addressed to fully evaluate the long‐term benefit of AOMs in the real world.

Importantly, the review identified large gaps in the evidence base for AOMs in treating patients with obesity or overweight in real‐world practice, including few comparative effectiveness studies and a narrow range of reported outcomes. Real‐world studies are also affected by the same issues that plague RCTs in the obesity field with respect to untangling the interactions between adjunct lifestyle measures and AOMs. There is a clear need for more extensive and consistently designed real‐world studies, including pragmatic trials, that incorporate valid control and/or comparator groups, that examine more recently approved medications, and that more robustly account for the relative contributions of lifestyle interventions. Such studies can capture a broader range of outcomes, including cardiometabolic, economic, and patient‐reported measures. Strengthening the approach to RWE generation in obesity will help build a more accurate picture of the value of AOMs in routine clinical practice, especially as newer agents promising greater efficacy are on the horizon.

## CONFLICT OF INTEREST

N.N.A., J.L.P., and H.K. are full‐time employees and shareholders of Eli Lilly and Company (Lilly). S.R. and T.K.‐M. are employees of KMHO and received funding from Lilly for time spent conducting this research. Lilly and authors employed by Lilly are involved in obesity drug research.

## Supporting information


**TABLE S1** MEDLINE search string
**Table S2** Embase search string
**Table S3** Cochrane Central Register of Controlled Trials (CENTRAL)
**Table S4** NHS Economic Evaluation Database
**Table S5** Health Technology Assessment Database
