# Supplementary material for: Clinical outcomes associated with anti‐obesity medications in real‐world practice: A systematic literature review
Source: Obes Rev. 2021 Aug 22;22(11):e13326. doi: 10.1111/obr.13326 (PMC9285776; doi:10.1111/obr.13326)
Supplement: Supplementary file 1 — TABLE S1 MEDLINE search string Table S2 Embase search string Table S3 Cochrane Central Register of Controlled Trials (CENTRAL) Table S4 NHS Economic Evaluation Database Table S5 Health Technology Assessment Database [file OBR-22-0-s001.pdf]

Clinical outcomes associated with anti-obesity medications in real-world practice: a systematic literature review

Nadia N Ahmad | Susan Robinson | Tessa Kennedy-Martin | Jiat Ling Poon | Hong Kan

Correspondence: Hong Kan, PhD, Global Patient Outcomes and Real-World Evidence, Eli Lilly and Company, Lilly Corporate Center, Indianapolis, IN 46285, USA
Mobile: 317-954-9088; Email: kan_hongjun@lilly.com

CONTENTS

Search strategy 1
TABLE S1 MEDLINE search string 2
TABLE S2 Embase search string 7
TABLE S3 Cochrane Central Register of Controlled Trials (CENTRAL) 12
TABLE S4 NHS Economic Evaluation Database 17
TABLE S5 Health Technology Assessment Database 19
References 21

Search strategy

Search terms for the RWE concept included those related to 'RWE', patient databases, claims databases and registries, and terms related to observational studies. These terms were based on the Canadian Agency for Drugs and Technologies (CADTH) in Health RWE filter and the CADTH observational study filter (Kaunelis et al, 2018). In addition, observational study terms draw on the Scottish Intercollegiate Guidelines Network (SIGN) search filters (SIGN, 2020). The search strategy also excludes animal studies using a standard algorithm and excludes records indexed as editorial or news publications.

A base-case strategy was developed for MEDLINE and adapted to the other databases. Search strings for each database are provided in Tables S1 to S5 below.

TABLE S1 MEDLINE search string

1 exp Overweight/ (206802)

2 body weight/ or body weight changes/ (185354)

3 Weight Gain/ (30240)

4 Weight Loss/ (34314)

5 body mass index/ (119749)

6 (weigh$ or overweight$).ti,ab,kf. (1032039)

7 (obes$ or antiobes$).ti,ab,kf. (287538)

8 (adipos$ or corpulen$ or fat overload).ti,ab,kf. (102552)

9 (overeat$ or over-eat$).ti,ab,kf. (2994)

10 (overfeed$ or over-feed$).ti,ab,kf. (1933)

11 (body mass or bmi or bmis).ti,ab,kf. (256862)

12 Appetite/ or Appetite Regulation/ or exp Satiation/ (14390)

13 appetite depressants/ or anti-obesity agents/ (8788)

14 appetite$.ti,ab,kf. (25166)

15 satiat$.ti,ab,kf. (3888)

16 or/1-15 (1527459)

17 Orlistat/ (1213)

18 orlistat$.ti,ab,kf,rn,nm. (1913)

19 (alli or allir or allitm or orlipastat$2 or ro 18 0647$2 or ro 180647$2 or ro180647$2 or ro 18 0647002$2 or ro 180647002$2 or ro180647002$2 or tetrahydrolipastatin$2 or tetrahydrolipstatin$2 or tetrahydro-lipastatin$2 or tetrahydro-lipstatin$2 or tetralipstatin$2 or tetra-lipstatin$2 or tetralipastatin$2 or tetra-lipastatin$2 or thlp$2 or xenical$2).ti,ab,kf,rn,nm. (600)

20 (95M8R751W8 or 96829-58-2).ti,ab,kf,rn,nm. (1214)

21 or/17-20 (2302)

22 lorcaserin$.ti,ab,kf,rn,nm. (376)

23 (apd 3 56$2 or apd-356$2 or apd356$2 or belviq$2 or lorqess$2).ti,ab,kf,rn,nm. (48)

24 (0qjf08gdpe or 3423ke0k4y or 616202-92-7 or 637e494o0z or 846589-98-8 or 856681-05-5 or rev26sr2b4).ti,ab,kf,rn,nm. (203)

25 or/22-24 (384)

26 Phentermine/ (789)

27 phentermin$.ti,ab,kf,rn,nm. (1104)

28 (0K2I505OTV or 1197-21-3 or 122-09-8 or 204-522-1 or 214-821-9 or C045TQL4WP).ti,ab,kf,rn,nm. (789)

29 (phenterminetopiramate$ or topiramatephentermin$).ti,ab,kf,rn,nm. (0)

30 (adipex$2 or adipexp$2 or beta aminoisopropylbenzene$2 or beta phenyltert butylamine$2 or beta phenyltertbutylamine$2 or dimethylphenethylamine$2 or dimethylphenetylamine$2 or diminex$2 or duromine$2 or "ex adipos$2" or exadipos$2 or fastin or fastinr or fastintm or ionamin$2 or ionamine$2 or ionomin$2 or linyl$2 or lomaira$2 or minobese-forte$2 or mirapront$2 or novirasin$2 or nsc44090$2 or nsc-44090$2 or obermine$2 or obestin30$2 or obestin-30$2 or obycap$2 or oby-cap$2 or obytrim$2 or oby-trim$2 or onamast$2 or ona-mast$2 or panbesy$2 or panbesyl$2 or phentercot$2 or phentermide$2 or phentrol$2 or profast$2 or pro-fast$2 or qnexa$2 or qsiva$2 or qsymia$2 or reducyl$2 or redusa$2 or sinpet$2 or suprenza$2 or tdiet$2 or t-diet$2 or terbutylamine$2 or tora or torar or toratm or umine$2 or wilpo$2 or zantryl$2).ti,ab,kf,rn,nm. (458)

31 or/26-30 (1504)

32 Naltrexone/ (7629)

33 naltrexon$.ti,ab,kf,rn,nm. (9782)

34 (antaxon$2 or antaxone$2 or celupan$2 or en-1639a$2 or en1639a$2 or nalerona$2 or nalorex$2 or naltrel$2 or nemexin$2 or nodict$2 or nutrexon$2 or phaltrexia$2 or regental$2 or revez$2 or revia$2 or re-via$2 or trexan$2 or vivitrex$2 or vivitrol$2).ti,ab,kf,rn,nm. (128)

35 (16590-41-3 or 16676-29-2 or 240-649-9 or 240-723-0 or 5P80UKS30B or 5S6W795CQM or 850808-02-5 or Z6375YW9SF).ti,ab,kf,rn,nm. (7631)
36 or/32-35 (9805)
37 Bupropion/ (2951)
38 bupropion$.ti,ab,kf,rn,nm. (4807)
39 (amfebutamon$ or aplenzin$2 or budeprion$2 or buprion$2 or bupropin$2 or buproprion$2 or buxon$2 or bw-323$2 or bw-323u66$2 or bw323$2 or bw323u66$2 or forfivo$2 or nsc-315851$2 or nsc315851$2 or odranal$2 or quomen$2 or wellbatrin$2 or wellbutrin$2 or zyban$2 or zyntabac$2).ti,ab,kf,rn,nm. (295)
40 (01ZG3TPX31 or 250-759-9 or 31677-93-7 or 324548-43-8 or 34911-55-2 or 437723-96-1 or 69BL03TL5X or 8KZL6AO7H4 or ZG7E5POY8O).ti,ab,kf,rn,nm. (2952)
41 or/37-40 (4879)
42 36 and 41 (261)
43 (contrave$2 or mysimba$2).ti,ab,kf,rn,nm. (247)
44 (bupropionnaltrexon$ or naltrexonebupropion$).ti,ab,kf,rn,nm. (0)
45 or/42-44 (473)
46 Liraglutide/ (1476)
47 liraglutide$.ti,ab,kf,rn,nm. (2607)
48 (ideglira$2 or nn-2211$2 or nn2211$2 or nn-9068$2 or nn9068$2 or nn-9924$2 or nn9924$2 or nnc-90-1170$2 or nnc90-1170$2 or saxenda$2 or victoza$2 or xultophy$2).ti,ab,kf,rn,nm. (188)
49 (204656-20-2 or 839I73S42A).ti,ab,kf,rn,nm. (1477)
50 or/46-49 (2626)
51 sibutramin$.ti,ab,kf,rn,nm. (1340)
52 (adisar$2 or arcalion$2 or atenix$2 or biomag$2 or bts 54 524$2 or bts 54524$2 or bts54 524$2 or bts54524$2 or butramin$2 or cf-alli$2 or ectiva$2 or medaria$2 or meridia or meridiar or meridiatm or nolipo$2 or raductil$2 or reductil$2 or reduten$2 or saciette$2 or sacietyl$2 or s#bus$2 or sibutral$2 or sibutrex$2 or sigran$2 or vazy$2).ti,ab,kf,rn,nm. (127)
53 (106650-56-0 or 125494-59-9 or 153341-22-1 or 153341-23-2 or 154752-44-0 or 154752-45-1 or 2k5e83h3ll or 439wn38xqx or 84485-00-7 or 9gg866zp1e or odb7rn3ovj or ogm0yhd1wf or wfw97v6hv0 or wv5ec51866).ti,ab,kf,rn,nm. (924)
54 or/51-53 (1420)
55 21 or 25 or 31 or 45 or 50 or 54 (7652)
56 16 and 55 (4590)
57 Databases, Factual/ (74341)
58 National practitioner data bank/ (340)
59 Databases as Topic/ (9071)
60 Geographic Information Systems/ (7480)
61 Health Information Systems/ (1173)
62 exp Medical Records/ (140953)
63 Datasets as Topic/ (3203)
64 exp Records as Topic/ (252472)
65 exp Registries/ (88914)
66 exp Drug Prescriptions/ (32247)
67 exp Drug Utilization/ (24043)
68 Database Management Systems/ (7588)
69 exp Data Mining/ (7867)
70 (administrative adj2 (claim$ or data$ or record$)).ti,ab,kf. (16424)
71 ((adverse drug reaction$ or ADR or ADRs) adj report$).ti,ab,kf. (1311)
72 big data$.ti,ab,kf. (5896)
73 ((claim or claims) adj2 data$).ti,ab,kf. (12990)
74 claims based.ti,ab,kf. (1092)

75 (data$ adj2 (links or linked or linkage$ or linking)).ti,ab,kf. (12625)
76 (data$ adj2 (mining or mine or mined)).ti,ab,kf. (10267)
77 (data$ adj2 repositor$).ti,ab,kf. (2459)
78 database management system$.ti,ab,kf. (515)
79 dataset.pt. (733)
80 discharge abstract$.ti,ab,kf. (865)
81 factual data$.ti,ab,kf. (129)
82 ((health or healthcare) adj3 (claim$ or data$ or record$)).ti,ab,kf. (71318)
83 (hospital$ adj2 (data$ or record$)).ti,ab,kf. (32030)
84 (inpatient$ adj3 (data$ or record$)).ti,ab,kf. (7355)
85 insurance.ti,ab,kf. (82864)
86 ((international or national or nationwide) adj3 data$).ti,ab,kf. (55684)
87 (managed care adj3 data$).ti,ab,kf. (477)
88 medical record$.ti,ab,kf. (101553)
89 medical transcription data$.ti,ab,kf. (1)
90 (medicare or medicaid).ti,ab,kf. (57244)
91 (nursing data$ or nursing record$).ti,ab,kf. (1225)
92 (patient$ adj3 (data$ or record$)).ti,ab,kf. (233296)
93 (billing adj3 (record$ or data$)).ti,ab,kf. (1914)
94 ((prescription$ or prescrib$) adj3 (claim$ or data$ or record$ or pattern$)).ti,ab,kf. (12155)
95 real world.ti,ab,kf. (35343)
96 (registries or registry).ti,ab,kf. (116632)
97 (register or registers).ti,ab,kf. (68118)
98 (routine$ adj5 data$).ti,ab,kf. (13736)
99 ((utilization or utilisation) adj3 (data$ or record$ or pattern$)).ti,ab,kf. (8880)
100 participant data$.ti,ab,kf. (1265)
101 real-life.ti,ab,kf. (16455)
102 physician claims.ti,ab,kf. (297)
103 (EMR or EMRs or EHR or EHRs or EPR or EPRs).ti,ab,kf. (35810)
104 ((institutional or multiinstitutional) adj2 data$).ti,ab,kf. (3475)
105 exp medicare/sn (5586)
106 medicaid/sn (3998)
107 or/57-106 (1079265)
108 Epidemiologic Methods/ (31104)
109 exp Epidemiologic Studies/ (2364131)
110 Observational Studies as Topic/ (4204)
111 Clinical Studies as Topic/ (385)
112 (Observational Study or Validation Studies or Clinical Study).pt. (166197)
113 (observational adj3 (study or studies or design or analysis or analyses)).ti,ab,kf. (131524)
114 cohort$.ti,ab,kf. (536968)
115 (prospective adj7 (study or studies or design or analysis or analyses)).ti,ab,kf. (401045)
116 ((follow up or followup) adj7 (study or studies or design or analysis or analyses)).ti,ab,kf. (129321)
117 longitudinal.ti,ab,kf. (231460)
118 ((longterm or (long adj term)) adj7 (study or studies or design or analysis or analyses or data)).ti,ab,kf. (116755)
119 retrospective.ti,ab,kf. (488420)
120 ((case adj control) or (case adj comparison) or (case adj controlled)).ti,ab,kf. (122650)
121 (case-referent adj3 (study or studies or design or analysis or analyses)).ti,ab,kf. (615)
122 (population adj3 (study or studies or analysis or analyses)).ti,ab,kf. (170394)
123 (descriptive adj3 (study or studies or design or analysis or analyses)).ti,ab,kf. (70284)

124 (cross adj sectional).ti,ab,kf. (322740)
125 ((natural adj experiment) or (natural adj experiments)).ti,ab,kf. (1937)
126 ((non experiment or nonexperiment or non experimental or nonexperimental) adj3 (study or studies or design or analysis or analyses)).ti,ab,kf. (1297)
127 case series.ti,ab,kf. (68165)
128 case reports.pt. (2046425)
129 (case adj3 (report or reports or study or studies or histories)).ti,ab,kf. (757454)
130 organizational case studies/ (12121)
131 or/108-130 (5499940)
132 Hospitalization/ (100918)
133 data collection/ (88230)
134 insurance/ or insurance claim reporting/ or "insurance claim review"/ or insurance, health/ or insurance, health, reimbursement/ (56181)
135 (dataset or datasets or data-set or data-sets).ti,ab,kf. (183861)
136 ((provider or payor or payer) adj data$).ti,ab,kf. (337)
137 (database adj (study or studies)).ti,ab,kf. (1895)
138 (reimbursement adj3 (data$ or record$)).ti,ab,kf. (845)
139 (medical chart or medical charts or chart review or chart reviews).ti,ab,kf. (44005)
140 ((utilization or utilisation) adj3 (drug$ or medicine$ or medication$)).ti,ab,kf. (5173)
141 ((digital or dark or coroner$ or pharmacy or pharmacies or "secondary use") adj3 data$).ti,ab,kf. (6866)
142 or/132-141 (476086)
143 Pragmatic Clinical Trials as Topic/ (339)
144 pragmatic clinical trial.pt. (1150)
145 (pragmatic adj (trial$ or study or studies or clinical trial$ or clinical study or clinical studies)).ti,ab,kf. (1415)
146 ((adaptive or bridging or practical or naturalistic or simple) adj (trial$ or study or studies or clinical trial$ or clinical study or clinical studies)).ti,ab,kf. (2890)
147 exp Product Surveillance, Postmarketing/ (14736)
148 (postmarket$ or post-market$).ti,ab,kf. (6610)
149 (non-randomised or nonrandomised or non-randomized or nonrandomized).ti,ab,kf. (23625)
150 or/143-149 (47577)
151 "surveys and questionnaires"/ or health care surveys/ or health surveys/ (508811)
152 (survey$ or questionnaire$).ti,ab,kf. (979411)
153 or/151-152 (1149776)
154 107 or 131 or 142 or 150 or 153 (6928583)
155 Economics/ (27074)
156 exp "costs and cost analysis"/ (228138)
157 Economics, Dental/ (1907)
158 exp economics, hospital/ (23863)
159 Economics, Medical/ (9031)
160 Economics, Nursing/ (3993)
161 Economics, Pharmaceutical/ (2889)
162 (economic$ or cost or costs or costly or costing or price or prices or pricing or pharmacoeconomic$).ti,ab. (741790)
163 (expenditure$ not energy).ti,ab. (28144)
164 value for money.ti,ab. (1599)
165 budget$.ti,ab. (27839)
166 or/155-165 (890141)
167 ((energy or oxygen) adj cost).ti,ab. (3949)
168 (metabolic adj cost).ti,ab. (1342)

169	((energy or oxygen) adj expenditure).ti,ab. (23955)
170	or/167-169 (28293)
171	166 not 170 (883624)
172	56 and 154 (1299)
173	56 and 171 (244)
174	or/172-173 (1452)
175	exp animals/ not humans/ (4617436)
176	(news or editorial).pt. (699936)
177	174 not (175 or 176) (1424)
178	limit 177 to english language (1339)
179	remove duplicates from 178 (1336)

TABLE S2 Embase search string

1 exp obesity/ (491271)
2 body weight/ or body weight change/ or body weight control/ or body weight fluctuation/ or body weight variation/ (277374)
3 body weight gain/ or weight gain/ (100348)
4 body weight loss/ or weight reduction/ (173686)
5 body mass/ (404840)
6 (weigh$ or overweight$).ti,ab,kw,dj. (1381359)
7 (obes$ or antiobes$).ti,ab,kw,dj. (433382)
8 (adipos$ or corpulen$ or fat overload).ti,ab,kw,dj. (139346)
9 (overeat$ or over-eat$).ti,ab,kw,dj. (3997)
10 (overfeed$ or over-feed$).ti,ab,kw,dj. (2480)
11 (body mass or bmi or bmis).ti,ab,kw,dj. (453518)
12 appetite/ or food intake/ or satiety/ or satiety response/ (142952)
13 anorexigenic agent/ or antiobesity agent/ (7787)
14 appetite$.ti,ab,kw,dj. (38011)
15 satiat$.ti,ab,kw,dj. (4714)
16 or/1-15 (2282348)
17 *tetrahydrolipstatin/ (1285)
18 orlistat$.ti,ab,kw,tn. (2873)
19 (alli or allir or allitm or orlipastat$2 or ro 18 0647$2 or ro 180647$2 or ro180647$2 or ro 18 0647002$2 or ro 180647002$2 or ro180647002$2 or tetrahydrolipastatin$2 or tetrahydrolipstatin$2 or tetrahydro-lipastatin$2 or tetrahydro-lipstatin$2 or tetralipstatin$2 or tetra-lipstatin$2 or tetralipastatin$2 or tetra-lipastatin$2 or thlp$2 or xenical$2).ti,ab,kw,tn. (1508)
20 (95M8R751W8 or 96829-58-2).ti,ab,kw,tn. (0)
21 or/17-20 (4011)
22 *lorcaserin/ or lorcaserin$.ti,ab,kw,tn. (553)
23 (apd 3 56$2 or apd-356$2 or apd356$2 or belviq$2 or lorqess$2).ti,ab,kw,tn. (278)
24 (0qjf08gdpe or 3423ke0k4y or 616202-92-7 or 637e494o0z or 846589-98-8 or 856681-05-5 or rev26sr2b4).ti,ab,kw,tn. (0)
25 or/22-24 (708)
26 *phentermine/ or *phentermine resin/ or *phentermine plus topiramate/ (1022)
27 phentermin$.ti,ab,kw,tn. (1017)
28 (0K2I505OTV or 1197-21-3 or 122-09-8 or 204-522-1 or 214-821-9 or C045TQL4WP).ti,ab,kw,tn. (0)
29 (phenterminetopiramate$ or topiramatephentermin$).ti,ab,kw,tn. (4)
30 (adipex$2 or adipexp$2 or beta aminoisopropylbenzene$2 or beta phenyltert butylamine$2 or beta phenyltertbutylamine$2 or dimethylphenethylamine$2 or dimethylphenetylamine$2 or diminex$2 or duromine$2 or "ex adipos$2" or exadipos$2 or fastin or fastinr or fastintm or ionamin$2 or ionamine$2 or ionomin$2 or linyl$2 or lomaira$2 or minobese-forte$2 or mirapront$2 or novirasin$2 or nsc44090$2 or nsc-44090$2 or obermine$2 or obestin30$2 or obestin-30$2 or obycap$2 or oby-cap$2 or obytrim$2 or oby-trim$2 or onamast$2 or ona-mast$2 or panbesy$2 or panbesyl$2 or phentercot$2 or phentermide$2 or phentrol$2 or profast$2 or pro-fast$2 or qnexa$2 or qsiva$2 or qsymia$2 or reducyl$2 or redusa$2 or sinpet$2 or suprenza$2 or tdiet$2 or t-diet$2 or terbutylamine$2 or tora or torar or toratm or umine$2 or wilpo$2 or zantryl$2).ti,ab,kw,tn. (1130)
31 or/26-30 (2410)
32 *naltrexone/ (6034)
33 naltrexon$.ti,ab,kw,tn. (8949)
34 (antaxon$2 or antaxone$2 or celupan$2 or en-1639a$2 or en1639a$2 or nalerona$2 or nalorex$2 or naltrel$2 or nemexin$2 or nodict$2 or nutrexon$2 or phaltrexia$2 or regental$2 or revez$2 or revia$2 or re-via$2 or trexan$2 or vivitrex$2 or vivitrol$2).ti,ab,kw,tn. (765)

35 (16590-41-3 or 16676-29-2 or 240-649-9 or 240-723-0 or 5P80UKS30B or 5S6W795CQM or 850808-02-5 or Z6375YW9SF).ti,ab,kw,tn. (1)
36 or/32-35 (10554)
37 *amfebutamone/ (3397)
38 bupropion$.ti,ab,kw,tn. (6204)
39 (amfebutamon$ or aplenzin$2 or budeprion$2 or buprion$2 or bupropin$2 or buproprion$2 or buxon$2 or bw-323$2 or bw-323u66$2 or bw323$2 or bw323u66$2 or forfivo$2 or nsc-315851$2 or nsc315851$2 or odranal$2 or quomen$2 or wellbatrin$2 or wellbutrin$2 or zyban$2 or zyntabac$2).ti,ab,kw,tn. (2683)
40 (01ZG3TPX31 or 250-759-9 or 31677-93-7 or 324548-43-8 or 34911-55-2 or 437723-96-1 or 69BL03TL5X or 8KZL6AO7H4 or ZG7E5POY8O).ti,ab,kw,tn. (1)
41 or/37-40 (8638)
42 36 and 41 (454)
43 *amfebutamone plus naltrexone/ or (contrave$2 or mysimba$2).ti,ab,kw,tn. (534)
44 (bupropionnaltrexon$ or naltrexonebupropion$).ti,ab,kw,tn. (2)
45 or/42-44 (869)
46 *liraglutide/ (3036)
47 liraglutide$.ti,ab,kw,tn. (4991)
48 (ideglira$2 or nn-2211$2 or nn2211$2 or nn-9068$2 or nn9068$2 or nn-9924$2 or nn9924$2 or nnc-90-1170$2 or nnc90-1170$2 or saxenda$2 or victoza$2 or xultophy$2).ti,ab,kw,tn. (1118)
49 (204656-20-2 or 839I73S42A).ti,ab,kw,tn. (0)
50 or/46-49 (5677)
51 *sibutramine/ or sibutramin$.ti,ab,kw,tn. (1965)
52 (adisar$2 or arcalion$2 or atenix$2 or biomag$2 or bts 54 524$2 or bts 54524$2 or bts54 524$2 or bts54524$2 or butramin$2 or cf-alli$2 or ectiva$2 or medaria$2 or meridia or meridiar or meridiatm or nolipo$2 or raductil$2 or reductil$2 or reduten$2 or saciette$2 or sacietyl$2 or s#bus$2 or sibutral$2 or sibutrex$2 or sigran$2 or vazy$2).ti,ab,kw,tn. (847)
53 (106650-56-0 or 125494-59-9 or 153341-22-1 or 153341-23-2 or 154752-44-0 or 154752-45-1 or 2k5e83h3ll or 439wn38xqx or 84485-00-7 or 9gg866zp1e or odb7rn3ovj or ogm0yhd1wf or wfw97v6hv0 or wv5ec51866).ti,ab,kw,tn. (1)
54 or/51-53 (2500)
55 21 or 25 or 31 or 45 or 50 or 54 (13837)
56 16 and 55 (9233)
57 factual database/ (22615)
58 National Practitioner Data Bank/ (309)
59 data base/ (234817)
60 geographic information system/ (9831)
61 medical information system/ (20278)
62 exp medical record/ (231194)
63 "administrative claims (health care)"/ (454)
64 information processing/ (229682)
65 exp registration/ (194108)
66 prescription/ or drug substitution/ or "off label drug use"/ (230091)
67 drug utilization/ or "drug utilization review"/ (19951)
68 database management system/ (384)
69 data mining/ (11935)
70 (administrative adj2 (claim$ or data$ or record$)).ti,ab,kw,dj. (25323)
71 ((adverse drug reaction$ or ADR or ADRs) adj report$).ti,ab,kw,dj. (2979)
72 big data$.ti,ab,kw,dj. (7282)
73 ((claim or claims) adj2 data$).ti,ab,kw,dj. (24420)
74 claims based.ti,ab,kw,dj. (2086)

75 (data$ adj2 (links or linked or linkage$ or linking)).ti,ab,kw,dj. (18152)
76 (data$ adj2 (mining or mine or mined)).ti,ab,kw,dj. (13753)
77 (data$ adj2 repositor$).ti,ab,kw,dj. (3554)
78 database management system$.ti,ab,kw,dj. (713)
79 discharge abstract$.ti,ab,kw,dj. (1224)
80 factual data$.ti,ab,kw,dj. (189)
81 ((health or healthcare) adj3 (claim$ or data$ or record$)).ti,ab,kw,dj. (100098)
82 (hospital$ adj2 (data$ or record$)).ti,ab,kw,dj. (53197)
83 (inpatient$ adj3 (data$ or record$)).ti,ab,kw,dj. (14095)
84 insurance.ti,ab,kw,dj. (112582)
85 ((international or national or nationwide) adj3 data$).ti,ab,kw,dj. (84716)
86 (managed care adj3 data$).ti,ab,kw,dj. (825)
87 medical record$.ti,ab,kw,dj. (172217)
88 medical transcription data$.ti,ab,kw,dj. (6)
89 (medicare or medicaid).ti,ab,kw,dj. (78623)
90 (nursing data$ or nursing record$).ti,ab,kw,dj. (1463)
91 (patient$ adj3 (data$ or record$)).ti,ab,kw,dj. (432104)
92 (billing adj3 (record$ or data$)).ti,ab,kw,dj. (3360)
93 ((prescription$ or prescrib$) adj3 (claim$ or data$ or record$ or pattern$)).ti,ab,kw,dj. (22564)
94 real world.ti,ab,kw,dj. (59910)
95 (registries or registry).ti,ab,kw,dj. (198651)
96 (register or registers).ti,ab,kw,dj. (89581)
97 (routine$ adj5 data$).ti,ab,kw,dj. (20165)
98 ((utilization or utilisation) adj3 (data$ or record$ or pattern$)).ti,ab,kw,dj. (14086)
99 participant data$.ti,ab,kw,dj. (1738)
100 real-life.ti,ab,kw,dj. (29558)
101 physician claims.ti,ab,kw,dj. (452)
102 (EMR or EMRs or EHR or EHRs or EPR or EPRs).ti,ab,kw,dj. (48921)
103 ((institutional or multiinstitutional) adj2 data$).ti,ab,kw,dj. (7559)
104 medicare/ and statistics/ (3243)
105 medicaid/ and statistics/ (2236)
106 or/57-105 (1946503)
107 epidemiology/ (205159)
108 exp case control study/ or retrospective study/ or cohort analysis/ or longitudinal study/ or prospective study/ or cross-sectional study/ or follow up/ or family study/ or clinical study/ (3341938)
109 observational study/ (179453)
110 validation study/ (79767)
111 (observational adj3 (study or studies or design or analysis or analyses)).ti,ab,kw,dj. (208843)
112 cohort$.ti,ab,kw,dj. (919849)
113 (prospective adj7 (study or studies or design or analysis or analyses)).ti,ab,kw,dj. (598408)
114 ((follow up or followup) adj7 (study or studies or design or analysis or analyses)).ti,ab,kw,dj. (202934)
115 longitudinal.ti,ab,kw,dj. (313156)
116 ((longterm or (long adj term)) adj7 (study or studies or design or analysis or analyses or data)).ti,ab,kw,dj. (176473)
117 retrospective.ti,ab,kw,dj. (813196)
118 ((case adj control) or (case adj comparison) or (case adj controlled)).ti,ab,kw,dj. (161738)
119 (case-referent adj3 (study or studies or design or analysis or analyses)).ti,ab,kw,dj. (674)
120 (population adj3 (study or studies or analysis or analyses)).ti,ab,kw,dj. (256388)
121 (descriptive adj3 (study or studies or design or analysis or analyses)).ti,ab,kw,dj. (105870)

122 (cross adj sectional).ti,ab,kw,dj. (425475)
123 ((natural adj experiment) or (natural adj experiments)).ti,ab,kw,dj. (2172)
124 ((non experiment or nonexperiment or non experimental or nonexperimental) adj3 (study or studies or design or analysis or analyses)).ti,ab,kw,dj. (1657)
125 case series.ti,ab,kw,dj. (96137)
126 case report/ (2405451)
127 (case adj3 (report or reports or study or studies or histories)).ti,ab,kw,dj. (934666)
128 health services research/ (32882)
129 or/107-128 (7227823)
130 hospitalization/ (345533)
131 insurance/ or health insurance/ or reimbursement/ (202800)
132 (dataset or datasets or data-set or data-sets).ti,ab,kw,dj. (235037)
133 ((provider or payor or payer) adj data$).ti,ab,kw,dj. (648)
134 (database adj (study or studies)).ti,ab,kw,dj. (3540)
135 (reimbursement adj3 (data$ or record$)).ti,ab,kw,dj. (1575)
136 "medical record review"/ (118469)
137 (medical chart or medical charts or chart review or chart reviews).ti,ab,kw,dj. (87289)
138 ((utilization or utilisation) adj3 (drug$ or medicine$ or medication$)).ti,ab,kw,dj. (10626)
139 ((digital or dark or coroner$ or pharmacy or pharmacies or "secondary use") adj3 data$).ti,ab,kw,dj. (11079)
140 or/130-139 (927850)
141 pragmatic trial/ (532)
142 (pragmatic adj (trial$ or study or studies or clinical trial$ or clinical study or clinical studies)).ti,ab,kw,dj. (2030)
143 adaptive clinical trial/ (96)
144 "adaptive clinical trial (topic)"/ (34)
145 ((adaptive or bridging or practical or naturalistic or simple) adj (trial$ or study or studies or clinical trial$ or clinical study or clinical studies)).ti,ab,kw,dj. (4026)
146 exp postmarketing surveillance/ or "phase 4 clinical trial (topic)"/ or prescription drug monitoring program/ (37341)
147 (postmarket$ or post-market$).ti,ab,kw,dj. (11507)
148 (non-randomised or nonrandomised or non-randomized or nonrandomized).ti,ab,kw,dj. (33042)
149 or/141-148 (82494)
150 questionnaire/ or open ended questionnaire/ or structured questionnaire/ (667571)
151 health care survey/ or health survey/ (203545)
152 (survey$ or questionnaire$).ti,ab,kw,dj. (1337413)
153 or/150-152 (1531046)
154 106 or 129 or 140 or 149 or 153 (9420485)
155 Health Economics/ (32260)
156 exp Economic Evaluation/ (295652)
157 exp Health Care Cost/ (282184)
158 pharmacoeconomics/ (7122)
159 (econom$ or cost or costs or costly or costing or price or prices or pricing or pharmacoeconomic$).ti,ab. (995623)
160 (expenditure$ not energy).ti,ab. (38061)
161 (value adj2 money).ti,ab. (2309)
162 budget$.ti,ab. (36413)
163 or/155-162 (1249986)
164 (metabolic adj cost).ti,ab. (1439)
165 ((energy or oxygen) adj cost).ti,ab. (4152)

166 ((energy or oxygen) adj expenditure).ti,ab. (30542)
167 or/164-166 (35059)
168 163 not 167 (1242831)
169 56 and 154 (2901)
170 56 and 168 (723)
171 or/169-170 (3319)
172 (animal/ or animal experiment/ or animal model/ or animal tissue/ or nonhuman/) not exp human/ (5861503)
173 editorial.pt. (632782)
174 171 not (172 or 173) (3231)
175 limit 174 to english language (3069)
176 remove duplicates from 175 (2978)
177 (conference abstract or conference paper or conference proceeding or conference review).pt. (4342296)
178 176 not 177 (1974)
179 176 and 177 (1004)
180 limit 179 to yr="2018 -Current" (170)
181 178 or 180 (2144)

TABLE S3 Cochrane Central Register of Controlled Trials (CENTRAL)

#1 [mh Overweight] 14447
#2 [mh ^"body weight"] OR [mh ^"body weight changes"] 7906
#3 [mh ^"Weight Gain"] 2338
#4 [mh ^"Weight Loss"] 5450
#5 [mh ^"body mass index"] 9712
#6 (weigh* or overweight*) 122622
#7 (obes* or antiobes*) 40697
#8 (adipos* or corpulen* or "fat overload") 7098
#9 (overeat* or over next eat*) 448
#10 (overfeed* or over next feed*) 207
#11 ("body mass" or bmi or bmis) 63112
#12 [mh ^Appetite] OR [mh ^"Appetite Regulation"] OR [mh Satiation] 1954
#13 [mh ^"appetite depressants"] OR [mh ^"anti-obesity agents"] 837
#14 appetite* 9811
#15 satiat* 1239
#16 {or #1-#15} 172638
#17 [mh ^Orlistat] 242
#18 orlistat* 569
#19 (alli or allir or allitm or orlipastat* or ro next 18 next 0647* or ro next 180647* or ro180647* or ro next 18 next 0647002* or ro next 180647002* or ro180647002* or tetrahydrolipastatin* or tetrahydrolipstatin* or tetrahydro next lipastatin* or tetrahydro next lipstatin* or tetralipstatin* or tetra next lipstatin* or tetralipastatin* or tetra next lipastatin* or thlp* or xenical*):ti,ab,kw 163
#20 (95M8R751W8 or "96829-58-2") 3
#21 #17 or #18 or #19 or #20 613
#22 lorcaserin* 120
#23 (apd next 3 next 56* or apd next 356* or apd356* or belviq* or lorqess*) 15
#24 (0qjf08gdpe or 3423ke0k4y or "616202-92-7" or 637e494o0z or "846589-98-8" or "856681-05-5" or rev26sr2b4) 2
#25 #22 or #23 or #24 126
#26 [mh ^Phentermine] 86
#27 phentermin* 200
#28 (0K2I505OTV or "1197-21-3" or "122-09-8" or "204-522-1" or "214-821-9" or C045TQL4WP) 6
#29 (phenterminetopiramate* or topiramatephentermin*) 43
#30 (adipex* or adipexp* or beta next aminoisopropylbenzene* or beta next phenyltert next butylamine* or beta next phenyltertbutylamine* or dimethylphenethylamine* or dimethylphenetylamine* or diminex* or duromine* or ex next adipos* or exadipos* or fastin or fastinr or fastintm or ionamin* or ionamine* or ionomin* or linyl* or lomaira* or minobese next forte* or mirapront* or novirasin* or nsc44090* or nsc next 44090* or obermine* or obestin30* or obestin next 30* or obycap* or oby next cap* or obytrim* or oby next trim* or onamast* or ona next mast* or panbesy* or panbesyl* or phentercot* or phentermide* or phentrol* or profast* or pro next fast* or qnexa* or qsiva* or qsymia* or reducyl* or redusa* or sinpet* or suprenza* or tdiet* or t next diet* or terbutylamine* or tora or torar or toratm or umine* or wilpo* or zantryl*) 141
#31 #26 or #27 or #28 or #29 or #30 323
#32 [mh ^Naltrexone] 1129
#33 naltrexon* 2329

#34 (antaxon* or antaxone* or celupan* or en next 1639a* or en1639a* or nalerona* or nalorex* or naltrel* or nemexin* or nodict* or nutrexon* or phaltrexia* or regental* or revez* or revia* or re next via* or trexan* or vivitrex* or vivitrol*) 105
#35 ("16590-41-3" or "16676-29-2" or "240-649-9" or "240-723-0" or 5P80UKS30B or 5S6W795CQM or "850808-02-5" or Z6375YW9SF) 8
#36 #32 or #33 or #34 or #35 2341
#37 [mh ^Bupropion] 747
#38 bupropion* 1735
#39 (amfebutamon* or aplenzin* or budeprion* or buprion* or bupropin* or buproprion* or buxon* or bw next 323* or bw next 323u66* or bw323* or bw323u66* or forfivo* or nsc next 315851* or nsc315851* or odranal* or quomen* or wellbatrin* or wellbutrin* or zyban* or zyntabac*) 572
#40 (01ZG3TPX31 or "250-759-9" or "31677-93-7" or "324548-43-8" or "34911-55-2" or "437723-96-1" or 69BL03TL5X or 8KZL6AO7H4 or ZG7E5POY8O) 6
#41 #37 or #38 or #39 or #40 1930
#42 #36 and #41 112
#43 (contrave* or mysimba*) 64
#44 (bupropionnaltrexon* or naltrexonebupropion*) 50
#45 #42 or #43 or #44 170
#46 [mh ^Liraglutide] 511
#47 liraglutide* 1602
#48 (ideglira* or nn next 2211* or nn2211* or nn next 9068* or nn9068* or nn next 9924* or nn9924* or nnc next 90 next 1170* or nnc90 next 1170* or saxenda* or victoza* or xultophy*) 299
#49 ("204656-20-2" or 839I73S42A) 107
#50 #46 or #47 or #48 or #49 1610
#51 sibutramin* 386
#52 (adisar* or arcalion* or atenix* or biomag* or bts next 54 next 524* or bts next 54524* or bts54 next 524* or bts54524* or butramin* or cf next alli* or ectiva* or medaria* or meridia or meridiar or meridiatm or nolipo* or raductil* or reductil* or reduten* or saciette* or sacietyl* or s?bus* or sibutral* or sibutrex* or sigran* or vazy*) 107
#53 ("106650-56-0" or "125494-59-9" or "153341-22-1" or "153341-23-2" or "154752-44-0" or "154752-45-1" or 2k5e83h3ll or 439wn38xqx or "84485-00-7" or 9gg866zp1e or odb7rn3ovj or ogm0yhd1wf or wfw97v6hv0 or wv5ec51866) 3
#54 #51 or #52 or #53 472
#55 #21 or #25 or #31 or #45 or #50 or #54 3139
#56 #16 and #55 2345
#57 [mh ^"Databases, Factual"] 304
#58 [mh ^"National practitioner data bank"] 0
#59 [mh ^"Databases as Topic"] 45
#60 [mh ^"Geographic Information Systems"] 38
#61 [mh ^"Health Information Systems"] 11
#62 [mh "Medical Records"] 2518
#63 [mh ^"Datasets as Topic"] 13
#64 [mh "Records as Topic"] 4259
#65 [mh Registries] 910
#66 [mh "Drug Prescriptions"] 836
#67 [mh "Drug Utilization"] 491
#68 [mh ^"Database Management Systems"] 15
#69 [mh "Data Mining"] 19
#70 (administrative near/2 (claim* or data* or record*)) 1021
#71 ((adverse next drug next reaction* or ADR or ADRs) next report*) 212

#72 big next data* 117
#73 ((claim or claims) near/2 data*) 766
#74 "claims based" 62
#75 (data* near/2 (links or linked or linkage* or linking)) 588
#76 (data* near/2 (mining or mine or mined)) 145
#77 (data* near/2 repositor*) 113
#78 database next management next system* 29
#79 [mh ^dataset] 0
#80 (discharge next abstract*) 28
#81 (factual next data*) 96
#82 ((health or healthcare) near/3 (claim* or data* or record*)) 8829
#83 (hospital* near/2 (data* or record*)) 4179
#84 (inpatient* near/3 (data* or record*)) 430
#85 insurance 4833
#86 ((international or national or nationwide) near/3 data*) 2835
#87 ("managed care" near/3 data*) 33
#88 (medical next record*) 11053
#89 (medical next transcription next data*) 0
#90 (medicare or medicaid) 2779
#91 (nursing next data* or nursing next record*) 118
#92 (patient* near/3 (data* or record*)) 34262
#93 (billing near/3 (record* or data*)) 171
#94 ((prescription* or prescrib*) near/3 (claim* or data* or record* or pattern*)) 1341
#95 "real world" 5150
#96 (registries or registry) 44990
#97 (register or registers):ti,ab,kw 20946
#98 (routine* near/5 data*) 1745
#99 ((utilization or utilisation) near/3 (data* or record* or pattern*)) 1090
#100 (participant next data*) 759
#101 "real-life" 2665
#102 "physician claims" 7
#103 (EMR or EMRs or EHR or EHRs or EPR or EPRs) 1687
#104 ((institutional or multiinstitutional) near/2 data*) 196
#105 MeSH descriptor: [Medicare] explode all trees and with qualifier(s): [statistics & numerical data - SN] 49
#106 MeSH descriptor: [Medicaid] this term only and with qualifier(s): [statistics & numerical data - SN] 49
#107 {or #57-#106} 118612
#108 [mh ^"Epidemiologic Methods"] 828
#109 [mh "Epidemiologic Studies"] 149855
#110 [mh ^"Observational Studies as Topic"] 49
#111 [mh ^"Clinical Studies as Topic"] 3
#112 [mh ^"Observational Study"] OR [mh ^"Validation Studies"] OR [mh ^"Clinical Study"] 3
#113 (observational near/3 (study or studies or design or analysis or analyses)) 17621
#114 cohort* 58884
#115 (prospective near/7 (study or studies or design or analysis or analyses)) 182016
#116 (("follow up" or followup) near/7 (study or studies or design or analysis or analyses)) 126086
#117 longitudinal 19947
#118 ((longterm or "long term") near/7 (study or studies or design or analysis or analyses or data)) 27365

#119 retrospective 30835
#120 ("case control" or "case comparison" or "case controlled") 11802
#121 ("case-referent" near/3 (study or studies or design or analysis or analyses)) 10
#122 (population near/3 (study or studies or analysis or analyses)) 24946
#123 (descriptive near/3 (study or studies or design or analysis or analyses)) 4053
#124 ("cross sectional") 16474
#125 ("natural experiment" or "natural experiments") 131
#126 (("non experiment" or nonexperiment or "non experimental" or nonexperimental) near/3 (study or studies or design or analysis or analyses)) 114
#127 "case series" 4122
#128 [mh ^"Case Reports"] 0
#129 (case near/3 (report or reports or study or studies or histories)) 26343
#130 [mh ^"organizational case studies"] 26
#131 {or #108-#130} 396038
#132 [mh ^Hospitalization] 4881
#133 [mh ^"data collection"] 1220
#134 [mh ^insurance] OR [mh ^"insurance claim reporting"] OR [mh ^"insurance claim review"] OR [mh ^"insurance, health"] OR [mh ^"insurance, health, reimbursement"] 250
#135 (dataset or datasets or "data-set" or "data-sets") 6935
#136 ((provider or payor or payer) next data*) 40
#137 (database next (study or studies)) 162
#138 (reimbursement near/3 (data* or record*)) 100
#139 ("medical chart" or "medical charts" or "chart review" or "chart reviews") 2406
#140 ((utilization or utilisation) near/3 (drug* or medicine* or medication*)) 1341
#141 ((digital or dark or coroner* or pharmacy or pharmacies or "secondary use") near/3 data*) 551
#142 {or #132-#141} 17503
#143 [mh ^"Pragmatic Clinical Trials as Topic"] 16
#144 [mh ^"pragmatic clinical trial"] 0
#145 (pragmatic next (trial* or study or studies or clinical next trial* or clinical next study or clinical next studies)) 2853
#146 ((adaptive or bridging or practical or naturalistic or simple) next (trial* or study or studies or clinical next trial* or clinical next study or clinical next studies)) 818
#147 [mh "Product Surveillance, Postmarketing"] 194
#148 (postmarket* or post next market*) 1577
#149 ("non-randomised" or nonrandomised or "non-randomized" or nonrandomized) 10406
#150 {or #143-#149} 15406
#151 [mh ^"surveys and questionnaires"] OR [mh ^"health care surveys"] OR [mh ^"health surveys"] 25181
#152 (survey* or questionnaire*) 138130
#153 #151 or #152 138130
#154 #107 or #131 or #142 or #150 or #153 546235
#155 [mh ^Economics] 43
#156 [mh "costs and cost analysis"] 9987
#157 [mh ^"Economics, Dental"] 1
#158 [mh "economics, hospital"] 684
#159 [mh ^"Economics, Medical"] 25
#160 [mh ^"Economics, Nursing"] 12
#161 [mh ^"Economics, Pharmaceutical"] 66
#162 (economic* or cost or costs or costly or costing) 78525
#163 (price or prices or pricing or pharmacoeconomic*) 7642

#164	(expenditure* not energy)	1962
#165	"value for money"	270
#166	budget*	1221
#167	{or #155-#166}	82815
#168	((energy or oxygen) next cost)	435
#169	(metabolic next cost)	122
#170	((energy or oxygen) next expenditure)	4199
#171	#168 or #169 or #170	4614
#172	#167 not #171	81999
#173	#56 and #154	797
#174	#56 and #172	144
#175	#173 or #174	838
#176	#175 in Trials	734

TABLE S4 NHS Economic Evaluation Database

1	MeSH DESCRIPTOR Overweight EXPLODE ALL TREES	1058
2	MeSH DESCRIPTOR body weight	218
3	MeSH DESCRIPTOR body weight changes	0
4	MeSH DESCRIPTOR Weight Gain	155
5	MeSH DESCRIPTOR Weight Loss	464
6	MeSH DESCRIPTOR body mass index	363
7	((weigh* or overweight*))	8994
8	((obes* or antiobes*))	1520
9	((adipos* or corpulen* or fat overload))	95
10	((overeat* or over-eat*))	2
11	((overfeed* or over-feed*))	2
12	((body mass or bmi or bmis))	1191
13	MeSH DESCRIPTOR Appetite	12
14	MeSH DESCRIPTOR Appetite Regulation	1
15	MeSH DESCRIPTOR Satiation EXPLODE ALL TREES	9
16	MeSH DESCRIPTOR appetite depressants	34
17	MeSH DESCRIPTOR anti-obesity agents	74
18	(appetite*)	130
19	(satiat*)	8
20	#1 OR #2 OR #3 OR #4 OR #5 OR #6 OR #7 OR #8 OR #9 OR #10 OR #11 OR #12 OR #13 OR #14 OR #15 OR #16 OR #17 OR #18 OR #19	9922
21	(orlistat*)	69
22	((alli or allir or allitm or orlipastat* or ro 18 0647* or ro 180647* or ro180647* or ro 18 0647002* or ro 180647002* or ro180647002* or tetrahydrolipastatin* or tetrahydrolipstatin* or tetrahydro-lipastatin* or tetrahydro-lipstatin* or tetralipstatin* or tetra-lipstatin* or tetralipastatin* or tetra-lipastatin* or thlp* or xenical*))	6
23	((95M8R751W8 or 96829-58-2))	0
24	#21 OR #22 OR #23	69
25	(lorcaserin*)	3
26	((apd 3 56* or apd-356* or apd356* or belviq* or lorqess*))	1
27	((0qjf08gdpe or 3423ke0k4y or 616202-92-7 or 637e494o0z or 846589-98-8 or 856681-05-5 or rev26sr2b4))	0
28	#25 OR #26 OR #27	3
29	MeSH DESCRIPTOR Phentermine	6
30	(phentermin*)	9
31	((0K2I505OTV or 1197-21-3 or 122-09-8 or 204-522-1 or 214-821-9 or C045TQL4WP))	0
32	((phenterminetopiramate* or topiramatephentermin*))	0
33	((adipex* or adipexp* or beta aminoisopropylbenzene* or beta phenyltert butylamine* or beta phenyltertbutylamine* or dimethylphenethylamine* or dimethylphenetylamine* or diminex* or duromine* or "ex adipos*" or exadipos* or fastin or fastinr or fastintm or ionamin* or ionamine* or ionomin* or linyl* or lomaira* or minobese-forte* or mirapront* or novirasin* or nsc44090* or nsc-44090* or obermine* or obestin30* or obestin-30* or obycap* or oby-cap* or obytrim* or oby-trim* or onamast* or ona-mast* or panbesy* or panbesyl* or phentercot* or phentermide* or phentrol* or profast* or pro-fast* or qnexa* or qsiva* or qsymia* or reducyl* or redusa* or sinpet* or suprenza* or tdiet* or t-diet* or terbutylamine* or tora or torar or toratm or umine* or wilpo* or zantryl*))	3
34	#29 OR #30 OR #31 OR #32 OR #33	9
35	MeSH DESCRIPTOR Naltrexone	53
36	(naltrexon*)	97

37 ((antaxon* or antaxone* or celupan* or en-1639a* or en1639a* or nalerona* or nalorex* or naltrel* or nemexin* or nodict* or nutrexon* or phaltrexia* or regental* or revez* or revia* or re-via* or trexan* or vivitrex* or vivitrol*)) 2
38 ((16590-41-3 or 16676-29-2 or 240-649-9 or 240-723-0 or 5P80UKS30B or 5S6W795CQM or 850808-02-5 or Z6375YW9SF)) 0
39 #35 OR #36 OR #37 OR #38 97
40 MeSH DESCRIPTOR Bupropion 74
41 (bupropion*) 188
42 ((amfebutamon* or aplenzin* or budeprion* or buprion* or bupropin* or buproprion* or buxon* or bw-323* or bw-323u66* or bw323* or bw323u66* or forfivo* or nsc-315851* or nsc315851* or odranal* or quomen* or wellbatrin* or wellbutrin* or zyban* or zyntabac*)) 16
43 ((01ZG3TPX31 or 250-759-9 or 31677-93-7 or 324548-43-8 or 34911-55-2 or 437723-96-1 or 69BL03TL5X or 8KZL6AO7H4 or ZG7E5POY8O)) 0
44 #40 OR #41 OR #42 OR #43 194
45 #39 AND #44 5
46 ((contrave* or mysimba*)) 3
47 ((bupropionnaltrexon* or naltrexonebupropion*)) 0
48 #45 OR #46 OR #47 7
49 MeSH DESCRIPTOR Liraglutide 36
50 (liraglutide*) 58
51 ((ideglira* or nn-2211* or nn2211* or nn-9068* or nn9068* or nn-9924* or nn9924* or nnc-90-1170* or nnc90-1170* or saxenda* or victoza* or xultophy*)) 5
52 ((204656-20-2 or 839I73S42A)) 0
53 #49 OR #50 OR #51 OR #52 58
54 (sibutramin*) 58
55 ((adisar* or arcalion* or atenix* or biomag* or bts 54 524* or bts 54524* or bts54 524* or bts54524* or butramin* or cf-alli* or ectiva* or medaria* or meridia or meridiar or meridiatm or nolipo* or raductil* or reductil* or reduten* or saciette* or sacietyl* or s#bus* or sibutral* or sibutrex* or sigran* or vazy*)) 3
56 ((106650-56-0 or 125494-59-9 or 153341-22-1 or 153341-23-2 or 154752-44-0 or 154752-45-1 or 2k5e83h3ll or 439wn38xqx or 84485-00-7 or 9gg866zp1e or odb7rn3ovj or ogm0yhd1wf or wfw97v6hv0 or wv5ec51866)) 0
57 #54 OR #55 OR #56 60
58 #24 OR #28 OR #34 OR #48 OR #53 OR #57 159
59 (#20 and #58) 117
60 (#20 and #58) IN NHSEED 23

TABLE S5 Health Technology Assessment Database

1	MeSH DESCRIPTOR Overweight EXPLODE ALL TREES	1058
2	MeSH DESCRIPTOR body weight	218
3	MeSH DESCRIPTOR body weight changes	0
4	MeSH DESCRIPTOR Weight Gain	155
5	MeSH DESCRIPTOR Weight Loss	464
6	MeSH DESCRIPTOR body mass index	363
7	((weigh* or overweight*))	8994
8	((obes* or antiobes*))	1520
9	((adipos* or corpulen* or fat overload))	95
10	((overeat* or over-eat*))	2
11	((overfeed* or over-feed*))	2
12	((body mass or bmi or bmis))	1191
13	MeSH DESCRIPTOR Appetite	12
14	MeSH DESCRIPTOR Appetite Regulation	1
15	MeSH DESCRIPTOR Satiation EXPLODE ALL TREES	9
16	MeSH DESCRIPTOR appetite depressants	34
17	MeSH DESCRIPTOR anti-obesity agents	74
18	(appetite*)	130
19	(satiat*)	8
20	#1 OR #2 OR #3 OR #4 OR #5 OR #6 OR #7 OR #8 OR #9 OR #10 OR #11 OR #12 OR #13 OR #14 OR #15 OR #16 OR #17 OR #18 OR #19	9922
21	(orlistat*)	69
22	((alli or allir or allitm or orlipastat* or ro 18 0647* or ro 180647* or ro180647* or ro 18 0647002* or ro 180647002* or ro180647002* or tetrahydrolipastatin* or tetrahydrolipstatin* or tetrahydro-lipastatin* or tetrahydro-lipstatin* or tetralipstatin* or tetra-lipstatin* or tetralipastatin* or tetra-lipastatin* or thlp* or xenical*))	6
23	((95M8R751W8 or 96829-58-2))	0
24	#21 OR #22 OR #23	69
25	(lorcaserin*)	3
26	((apd 3 56* or apd-356* or apd356* or belviq* or lorqess*))	1
27	((0qjf08gdpe or 3423ke0k4y or 616202-92-7 or 637e494o0z or 846589-98-8 or 856681-05-5 or rev26sr2b4))	0
28	#25 OR #26 OR #27	3
29	MeSH DESCRIPTOR Phentermine	6
30	(phentermin*)	9
31	((0K2I505OTV or 1197-21-3 or 122-09-8 or 204-522-1 or 214-821-9 or C045TQL4WP))	0
32	((phenterminetopiramate* or topiramatephentermin*))	0
33	((adipex* or adipexp* or beta aminoisopropylbenzene* or beta phenyltert butylamine* or beta phenyltertbutylamine* or dimethylphenethylamine* or dimethylphenetylamine* or diminex* or duromine* or "ex adipos*" or exadipos* or fastin or fastinr or fastintm or ionamin* or ionamine* or ionomin* or linyl* or lomaira* or minobese-forte* or mirapront* or novirasin* or nsc44090* or nsc-44090* or obermine* or obestin30* or obestin-30* or obycap* or oby-cap* or obytrim* or oby-trim* or onamast* or ona-mast* or panbesy* or panbesyl* or phentercot* or phentermide* or phentrol* or profast* or pro-fast* or qnexa* or qsiva* or qsymia* or reducyl* or redusa* or sinpet* or suprenza* or tdiet* or t-diet* or terbutylamine* or tora or torar or toratm or umine* or wilpo* or zantryl*))	3
34	#29 OR #30 OR #31 OR #32 OR #33	9
35	MeSH DESCRIPTOR Naltrexone	53
36	(naltrexon*)	97

37 ((antaxon* or antaxone* or celupan* or en-1639a* or en1639a* or nalerona* or nalorex* or naltrel* or nemexin* or nodict* or nutrexon* or phaltrexia* or regental* or revez* or revia* or re-via* or trexan* or vivitrex* or vivitrol*)) 2
38 ((16590-41-3 or 16676-29-2 or 240-649-9 or 240-723-0 or 5P80UKS30B or 5S6W795CQM or 850808-02-5 or Z6375YW9SF)) 0
39 #35 OR #36 OR #37 OR #38 97
40 MeSH DESCRIPTOR Bupropion 74
41 (bupropion*) 188
42 ((amfebutamon* or aplenzin* or budeprion* or buprion* or bupropin* or buproprion* or buxon* or bw-323* or bw-323u66* or bw323* or bw323u66* or forfivo* or nsc-315851* or nsc315851* or odranal* or quomen* or wellbatrin* or wellbutrin* or zyban* or zyntabac*)) 16
43 ((01ZG3TPX31 or 250-759-9 or 31677-93-7 or 324548-43-8 or 34911-55-2 or 437723-96-1 or 69BL03TL5X or 8KZL6AO7H4 or ZG7E5POY8O)) 0
44 #40 OR #41 OR #42 OR #43 194
45 #39 AND #44 5
46 ((contrave* or mysimba*)) 3
47 ((bupropionnaltrexon* or naltrexonebupropion*)) 0
48 #45 OR #46 OR #47 7
49 MeSH DESCRIPTOR Liraglutide 36
50 (liraglutide*) 58
51 ((ideglira* or nn-2211* or nn2211* or nn-9068* or nn9068* or nn-9924* or nn9924* or nnc-90-1170* or nnc90-1170* or saxenda* or victoza* or xultophy*)) 5
52 ((204656-20-2 or 839I73S42A)) 0
53 #49 OR #50 OR #51 OR #52 58
54 (sibutramin*) 58
55 ((adisar* or arcalion* or atenix* or biomag* or bts 54 524* or bts 54524* or bts54 524* or bts54524* or butramin* or cf-alli* or ectiva* or medaria* or meridia or meridiar or meridiatm or nolipo* or raductil* or reductil* or reduten* or saciette* or sacietyl* or s#bus* or sibutral* or sibutrex* or sigran* or vazy*)) 3
56 ((106650-56-0 or 125494-59-9 or 153341-22-1 or 153341-23-2 or 154752-44-0 or 154752-45-1 or 2k5e83h3ll or 439wn38xqx or 84485-00-7 or 9gg866zp1e or odb7rn3ovj or ogm0yhd1wf or wfw97v6hv0 or wv5ec51866)) 0
57 #54 OR #55 OR #56 60
58 #24 OR #28 OR #34 OR #48 OR #53 OR #57 159
59 (#20 and #58) 117
60 (#20 and #58) IN NHSEED 23
61 (#20 and #58) IN HTA 24

References

Kaunelis D. Searching the literature for real world evidence. HTAi Annual Meeting, 2018. Vancouver, Canada.

Scottish Intercollegiate Guideline Network (SIGN). Search filters. Available at: https://www.sign.ac.uk/what-we-do/methodology/search-filters/ (accessed 30 Sep 2020).
